# The salivary microbiome as a diagnostic biomarker of periodontitis: a 16S multi-batch study before and after the removal of batch effects

**DOI:** 10.3389/fcimb.2024.1405699

**Published:** 2024-07-12

**Authors:** Alba Regueira-Iglesias, Berta Suárez-Rodríguez, Triana Blanco-Pintos, Marta Relvas, Manuela Alonso-Sampedro, Carlos Balsa-Castro, Inmaculada Tomás

**Affiliations:** ^1^ Oral Sciences Research Group, Special Needs Unit, Department of Surgery and Medical-Surgical Specialties, School of Medicine and Dentistry, Instituto de Investigación Sanitaria de Santiago (IDIS), Universidade de Santiago de Compostela, Santiago de Compostela, Spain; ^2^ Instituto Universitário de Ciências da Saúde, Cooperativa de Ensino Superior Politécnico e Universitário (IUCS-CESPU), Unidade de Investigação em Patologia e Reabilitação Oral (UNIPRO), Gandra, Portugal; ^3^ Department of Internal Medicine and Clinical Epidemiology, Instituto de Investigación Sanitaria de Santiago (IDIS), Complejo Hospitalario Universitario, Santiago de Compostela, Spain

**Keywords:** periodontal diseases, saliva, microbiome, 16S rRNA gene, next-generation sequencing, batch effects, differential abundance, predictive modeling

## Abstract

**Introduction:**

Microbiome-based clinical applications that improve diagnosis related to oral health are of great interest to precision dentistry. Predictive studies on the salivary microbiome are scarce and of low methodological quality (low sample sizes, lack of biological heterogeneity, and absence of a validation process). None of them evaluates the impact of confounding factors as batch effects (BEs). This is the first 16S multi-batch study to analyze the salivary microbiome at the amplicon sequence variant (ASV) level in terms of differential abundance and machine learning models. This is done in periodontally healthy and periodontitis patients before and after removing BEs.

**Methods:**

Saliva was collected from 124 patients (50 healthy, 74 periodontitis) in our setting. Sequencing of the V3-V4 16S rRNA gene region was performed in Illumina MiSeq. In parallel, searches were conducted on four databases to identify previous Illumina V3-V4 sequencing studies on the salivary microbiome. Investigations that met predefined criteria were included in the analysis, and the own and external sequences were processed using the same bioinformatics protocol. The statistical analysis was performed in the R-Bioconductor environment.

**Results:**

The elimination of BEs reduced the number of ASVs with differential abundance between the groups by approximately one-third (Before=265; After=190). Before removing BEs, the model constructed using all study samples (796) comprised 16 ASVs (0.16%) and had an area under the curve (AUC) of 0.944, sensitivity of 90.73%, and specificity of 87.16%. The model built using two-thirds of the specimens (training=531) comprised 35 ASVs (0.36%) and had an AUC of 0.955, sensitivity of 86.54%, and specificity of 90.06% after being validated in the remaining one-third (test=265). After removing BEs, the models required more ASVs (all samples=200–2.03%; training=100–1.01%) to obtain slightly lower AUC (all=0.935; test=0.947), lower sensitivity (all=81.79%; test=78.85%), and similar specificity (all=91.51%; test=90.68%).

**Conclusions:**

The removal of BEs controls false positive ASVs in the differential abundance analysis. However, their elimination implies a significantly larger number of predictor taxa to achieve optimal performance, creating less robust classifiers. As all the provided models can accurately discriminate health from periodontitis, implying good/excellent sensitivities/specificities, the salivary microbiome demonstrates potential clinical applicability as a precision diagnostic tool for periodontitis.

## 1 Introduction

Next-generation sequencing (NGS) studies of the 16S rRNA gene are characterized by heterogeneity in the results of salivary microbiota present in different periodontal conditions (Balan et al., 2018; Lu et al., 2022; Suzuki et al., 2022). This has resulted in a proliferation of narrative reviews that seek to define consensus microbial profiles associated with health or diseases (Belstrøm, 2020). However, methodological differences in the original research concerning the relevant steps of the 16S NGS workflow significantly affect the results of bacterial diversity obtained. This makes comparisons in the form of traditional reviews controversial (de la Cuesta-Zuluaga and Escobar, 2016; Robinson et al., 2016; Nearing et al., 2021).

Sequencing technologies perform differently regarding the read length, sequence throughput, and error rate (de la Cuesta-Zuluaga and Escobar, 2016), with Illumina performing better than Roche 454 or Ion Torrent (Nearing et al., 2021). Moreover, as demonstrated *in silico* using primer pairs with coverage values ≥90%, the oral species detected when amplifying a given gene region tend not to be covered when another zone is targeted, and vice versa (Regueira-Iglesias et al., 2023b). Thus, comparing or analyzing sequences or microbial diversity data obtained using different sequencing technologies and gene regions is problematic.

Additionally, the problems associated with using both the clustering of operational taxonomic units (OTU) (Regueira Iglesias et al., 2022) and phylogenetically diverse databases for taxonomic assignment are well known (Soergel et al., 2012; Edgar, 2018). However, these approaches have been used in approximately 70% of publications on the salivary microbiota present in different periodontal conditions in the last five years. In this respect, comparing diversity data obtained with different pipelines and databases is highly questionable (Nearing et al., 2021; Zaura et al., 2021).

Consequently, studies using denoising methods, which are considered more reliable (e.g. amplicon sequence variants - ASVs) (Callahan et al., 2017; Caruso et al., 2019; Prodan et al., 2020) as well as high-quality and specific oral databases are needed to achieve accurate taxonomic classifications (Escapa et al., 2020). On the other hand, microbiome data are characterized by their high-dimensional structured multivariate sparse data and their compositional nature (i.e., compositional data, CoDA) (Calle, 2019). Still, many investigators are unaware of this (Gloor et al., 2017), so the analyses performed in most of the oral microbiome studies did not consider its compositional nature (Chen et al., 2015; Balan et al., 2018; Damgaard et al., 2019; Lundmark et al., 2019; Sun et al., 2020; Ma et al., 2021; Lu et al., 2022; Suzuki et al., 2022).

Besides the technical factors already mentioned, there has been growing concern over the last few years about the influence of so-called batch effects (BEs). BEs include any sources of unwanted biological, technical, or computational variations that are unrelated to but obscure the biological element of interest (Wang and LêCao, 2020). Although microbiome-specific methods have been developed to remove BEs (Gibbons et al., 2018; Dai et al., 2019; Ling et al., 2022; Ma, 2022; Wang and Lê Cao, 2023), their use is not yet widespread. They have not been employed in any 16S rRNA gene sequencing research on salivary microbiota.

Potential microbiome-based clinical applications to improve prevention, diagnosis, or drug response related to oral and systemic health are of great interest for precision dentistry (Bourgeois et al., 2022; Zaura, 2022). Saliva has long been considered a fluid with predictive potential for health conditions, mainly due to the ease and non-invasiveness with which it can be collected and its abundance of biomarkers (Javaid et al., 2016; Kaczor-Urbanowicz et al., 2017). However, predictive analyses on oral microbiome data are challenging because they require very large and evenly distributed sample sizes between study groups, biological heterogeneity, and a validation process. To our knowledge, no salivary microbiome publication fulfills these mandatory methodological premises in developing generalizable predictive models (Kuhn and Johnson, 2013).

Given all of the above, we have conducted the present 16S multi-batch (16S-MB) study to provide the most robust evidence on the salivary microbial biomarkers for diagnosing both periodontal health and periodontal diseases. The study aimed to evaluate the salivary microbiota at the ASV level in relation to differential abundance and predictive models for distinct periodontal conditions before and after the removal of BEs under a CoDA analysis approach. Sequences stored in public repositories from earlier Illumina V3-V4 publications on the salivary microbiome were evaluated. We added to these further sequences derived from the saliva of periodontally healthy and periodontitis patients in our setting, which we obtained via the same platform and gene region. A unique bioinformatics protocol for high-quality filtering and sequence analysis was applied, employing an oral-specific database for the taxonomic assignment. Predictive models were built using all the samples and a subset of training specimens. The latter were subsequently validated with test samples.

## 2 Material and methods

**Figure 1:**
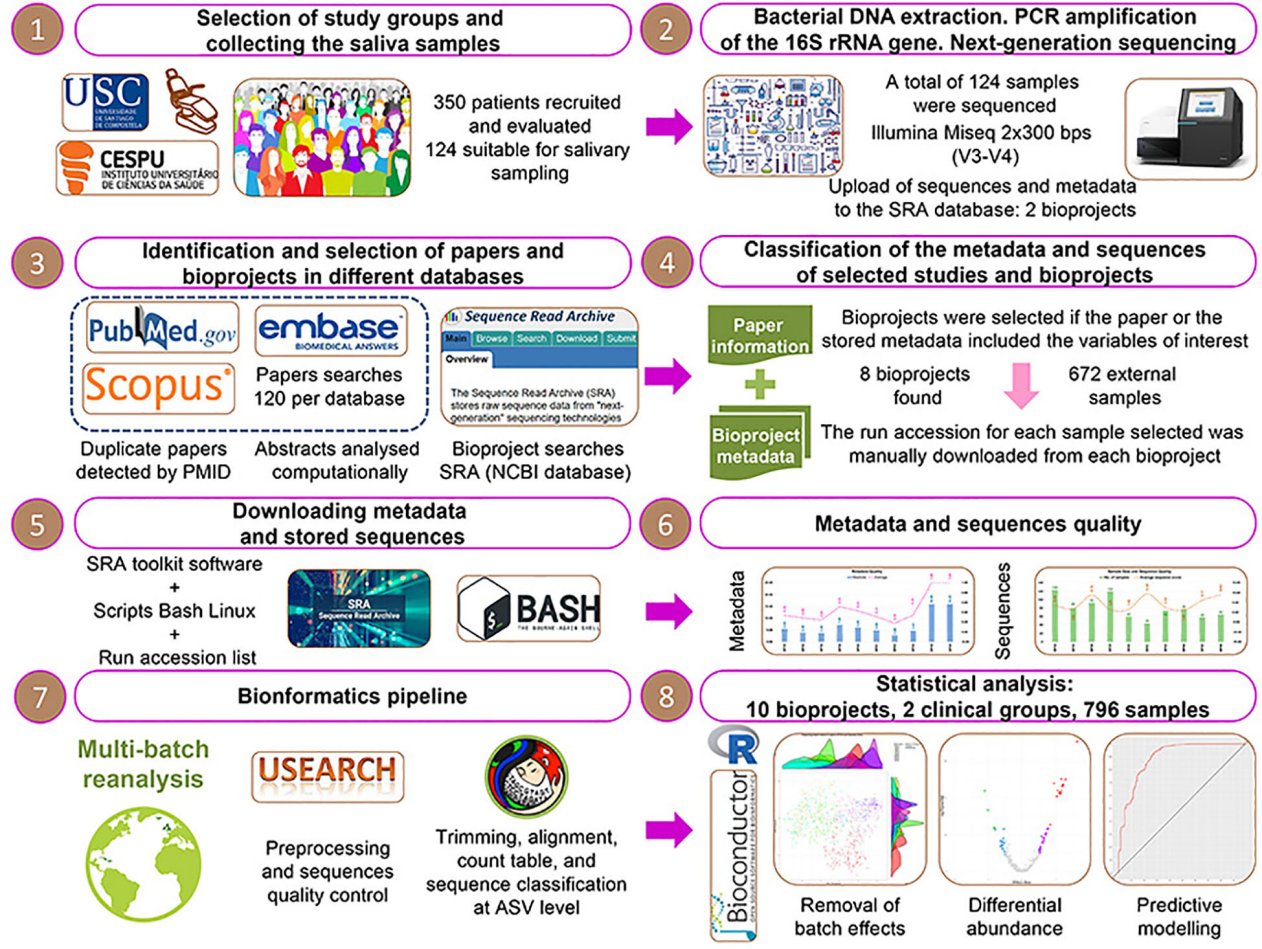
The complete protocol applied in the present multi-batch study.

The complete analysis protocol applied in the present study is represented in **Figure 1**.

### 2.1 Inclusion and exclusion criteria

The present investigation included studies on the salivary microbiota of adult patients with distinct periodontal health conditions. The V3-V4 region was targeted, and the Illumina sequencing technology was employed. The predefined standards for the articles in the literature and their metadata and sequences are included in **Data Sheet 1**.

### 2.2 Search methods for the identification and selection of investigations

The description of the search strategy and the manipulation of the data identified in the searches are included in **Data Sheet 1**. The terms used to perform the searches and to evaluate the articles found are included in **Data Sheet 2**.

The identifiers of the bioprojects from the selected investigations were used to access the information in the Sequence Read Archive (SRA) database (Leinonen et al., 2011) and the SRA run selector (https://www.ncbi.nlm.nih.gov/Traces/study/). At this point, our two bioprojects (PRJNA774299 and PRJNA774981) were added to the total. The information related to patients from our setting and the sequencing process of their samples is included in **Data Sheet 3**.

### 2.3 Classification of the information of the selected investigations

A new metadata table was constructed for each bioproject, and sequences were downloaded as described in **Data Sheet 1**.

### 2.4 Preprocessing and quality control of the sequences. Mothur pipeline

The preprocessing and quality assessments of the sequences were performed with USEARCH (Edgar, 2010), applying the filters indicated in **Data Sheet 1**. We employed the mothur pipeline (Schloss et al., 2009) for ASVs, with slight modifications that included using the Escapa et al. (2020) oral-specific database for taxonomic assignment. Sequences with >400 base pairs (bps) were allowed. We removed those with >8 homopolymers, regarded as chimeras by the VSEARCH algorithm (Rognes et al., 2016), and those classified as unknown taxa at the highest hierarchical level. Sequences were not clustered to any level, as we aimed to identify and classify the highest number of sequences possible at the ASV level. Finally, the count table, the taxonomic hierarchy at the ASV level, the phylogenetic tree, and the metadata table were exported to R-Bioconductor (Gentleman et al., 2004).

### 2.5 Assessment of the methodological quality of the selected investigations

The quality of the bioprojects’ metadata and sequences were evaluated as described in **Data Sheet 1**. The final number obtained representing the metadata quality was categorized as low= 0.00–0.33; medium= 0.34–0.66; and high= 0.67–1.00. The average sequence score (ASS) values were interpreted as very low-quantity= <0.25; low-quantity= 0.25–0.75; acceptable-quantity= 0.75–1.00; high-quantity= 1.00–2.00; and very high-quantity sequences= >2.00.

### 2.6 Statistical analysis with R-Bioconductor

The statistical analysis of the 16S rRNA gene sequencing data at the ASV level was performed using R (4.1.2) (R Core Team, 2022) and R-Bioconductor (Gentleman et al., 2004) to read the data and create a phyloseq object (phyloseq package 1.40.0) (McMurdie and Holmes, 2013). Samples with <2,500 sequences were excluded, leaving us with 814 specimens that were assigned to one of three groups according to the periodontal condition of the patients:

1) Saliva; periodontal health (Sal_x0Hxx= 483).
2) Saliva; gingivitis (Sal_x0Gxx= 18).
3) Saliva; untreated periodontitis (Sal_x0Pxx= 313).

The gingivitis group was removed due to its low sample size for developing predictive models, leaving 796 samples for analysis. ASVs with an abundance ≤10 counts and present in ≤2 samples were also excluded (Bourgon et al., 2010), meaning 9,859 ASVs remained.

We then converted the data from the phyloseq (McMurdie and Holmes, 2013) object-count matrix into percentage normalized data and applied the following abundance filters: 0.00%, 0.05%, 0.10%, and 0.20%. This meant that we obtained four different matrices in which the abundance of each taxon was above the set threshold in the total number of samples. The total ASVs and species for each filter were 9,859 and 573, 1,429 and 333, 659 and 208, and 355 and 142, respectively.

In parallel, an offset of one was added to the original count matrix (i.e., a value of one was added to all non-normalized data, all taxa) and a centered log-ratio (CLR) transformation was performed using the mixOmics package (6.22.0) (Rohart et al., 2017). Then, analyses were performed using the CLR-transformed data matrix, and for each of them, we ran each of the abundance filters first so that all the analyses were conducted for four abundance filters.

#### 2.6.1 Analysis for the elimination of BEs

**Figure 2:**
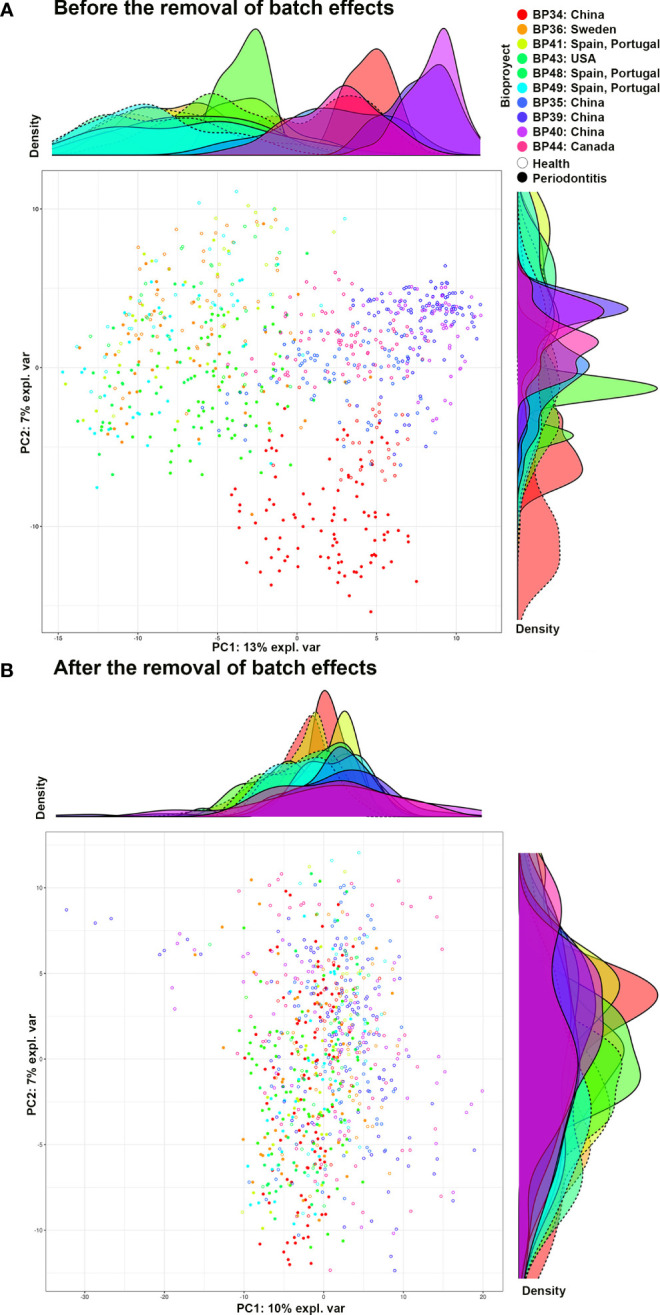
PCA and density graph representing the periodontally healthy and periodontitis samples of the different bioprojects. **(A)** Before removing the batch effects; and **(B)** After removing the batch effects. Graph **(A)** shows how, before eliminating batch effects, the samples from the same bioproject tend to be clustered together. In addition, groups of bioprojects are observed: 1) BP34 (China), 2) BP36, 41, 43, 48, and 49 (Spain and Portugal, Sweden, and the United States of America), and 3) BP35, 39, 40, and 44 (China and Canada). Graph **(B)**, after eliminating batch effects, shows the samples of the different bioprojects intermixed with each other, with no defined groups. BP34= Sun et al., 2020 - China; BP35= Ji et al., 2020 - China; BP36= Lundmark et al., 2019 - Sweden; BP39= Zhu et al., 2020b - China; BP40= Zhu et al., 2020a - China; BP41= Relvas et al., 2021 - Spain and Portugal; BP43= Annavajhala et al., 2020 - United States of America; BP44= Hall et al., 2017 - Canada; BP48= own unpublished data (PRJNA774299) - Spain and Portugal; BP49= own unpublished data (PRJNA774981) - Spain and Portugal.

BEs were analyzed as Wang and Lê Cao (2023) described, and the procedure is set out in **Data Sheet 1**. As a last step in this approach, BEs were removed using the following methods: 1) the removeBatchEffect function of the limma package (3.52.4) (Ritchie et al., 2015); 2) the ComBat function of the surrogate variable analysis package (3.44.0) (Leek et al., 2022); 3) a partial least-squares discriminant analysis (PLS-DA); 4) a sparse PLS-DA (sPLS-DA); 5) the percentile_norm functions of the PLSDA-batch package (Wang and Lê Cao, 2023); and 6) RUVIV of the remove unwanted variation package (0.9.7.1) (Gagnon-Bartsch, 2019). The performance of each method was evaluated, with removeBatchEffect (Ritchie et al., 2015) and ComBat (Leek et al., 2022) being the best for the different abundance filters (**Supplementary Figure 1**). The distribution of samples from subjects with periodontal health and periodontitis from the different bioprojects before and after removing the BEs was visualized using a principal component analysis (PCA) and density plot (**Figure 2**).

#### 2.6.2 Analysis of differential abundance

The mean difference between all the ASVs for both analysis groups was assessed using the non-parametric Mann-Whitney-Wilcoxon test. The p-value obtained was adjusted with the Benjamini-Hochberg correction using the mutoss package (0.1–13) (MuToss Coding Team et al., 2023). We got each ASV’s corresponding effect size, including its confidence interval and magnitude (large, medium, small, and negligible), using the Cohen’s d and Hedges’ g statistics from the effsize package (0.8.1) (Torchiano, 2020). ASVs with an adjusted p-value <0.01 were considered to have differential abundance.

#### 2.6.3 Predictive modeling analysis

The mixOmics package (Rohart et al., 2017) was used to conduct a supervised classification using an sPLS-DA (Lê Cao et al., 2011). This was done to facilitate categorizing the two clinical groups and identify the ASVs that best distinguished them. Predictive models were built, initially using all the study samples, and then, a subset of training specimens was subsequently validated with the remaining test samples. Taxa below each of the four abundance thresholds were excluded from the development of the models. The number of components in each model was determined by applying the rule of thumb K-1 (K= number of classes; here, two clinical groups). Receiver Operating Characteristic (ROC) curves were constructed with the true positivity rate (sensitivity) as a function of the false positivity rate (1-specificity). The following diagnostic performance parameters were calculated using the confusionMatrix function of the caret package (6.0–93) (Kuhn et al., 2023): area under the curve (AUC), accuracy (ACC), sensitivity, specificity, positive predictive value (PPV), and negative predictive value (NPV).

Finally, the number of predictor variables was reduced in the models obtained using the method above (i.e., best models): five by five up to 30, and one by one below 30, until we were left with only one ASV. Every diagnostic accuracy estimator was calculated for each number of predictors.

After evaluating the results obtained by the analyses using the four thresholds of abundance filtering, our focus was on describing the outcomes achieved by the high-abundance taxa (>0.20%).

## 3 Results

### 3.1 Investigations in the search process

**Figure 3:**
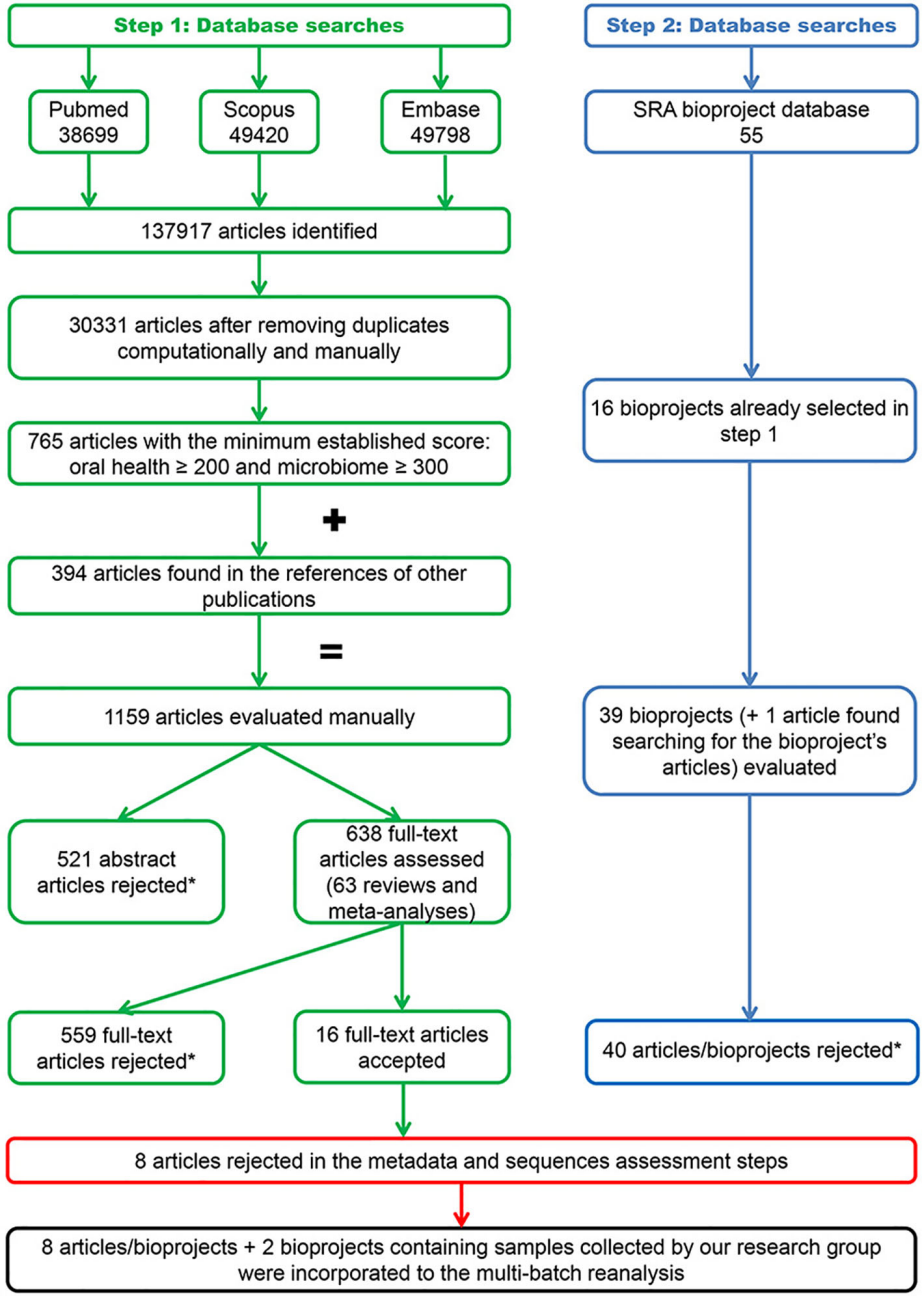
Flowchart of the search process. The exclusion reasons relating to the rejected articles and bioprojects are contained in **Data Sheets 4**–**6**.

**Figure 3** illustrates the flowchart of the search process. The 120 searches performed per electronic database identified 30,331 abstracts after the automatic and manual removal of duplicates. A total of 1,159 articles identified in these searches (**Data Sheet 4**) and 40 articles/bioprojects obtained from the SRA database (Leinonen et al., 2011) (**Data Sheet 5**) were selected for manual assessment. Ultimately, 16 articles with sequence data deposited in 16 bioprojects met the inclusion criteria (**Data Sheet 6**).

Six authors were contacted to obtain or clarify the metadata required to process the sequences, three of whom provided the information required. Five investigations were excluded after assessing the metadata quality and three after evaluating the samples and sequences. Consequently, the present 16S-MB study included eight articles (Hall et al., 2017; Lundmark et al., 2019; Annavajhala et al., 2020; Ji et al., 2020; Sun et al., 2020; Zhu et al., 2020a, Zhu et al., 2020b; Relvas et al., 2021) with sequence data deposited in eight bioprojects. We added to these bioprojects our information from patients recruited in our setting (two bioprojects). This produced a total of 796 samples, which were distributed in two clinical groups: Sal_x0Hxx (n= 313) and Sal_x0Pxx (n= 483). The main descriptive characteristics of the investigations included in the study are detailed in **Data Sheet 7**.

### 3.2 Quality assessment of the metadata and sequences from the included investigations

**Figure 4:**
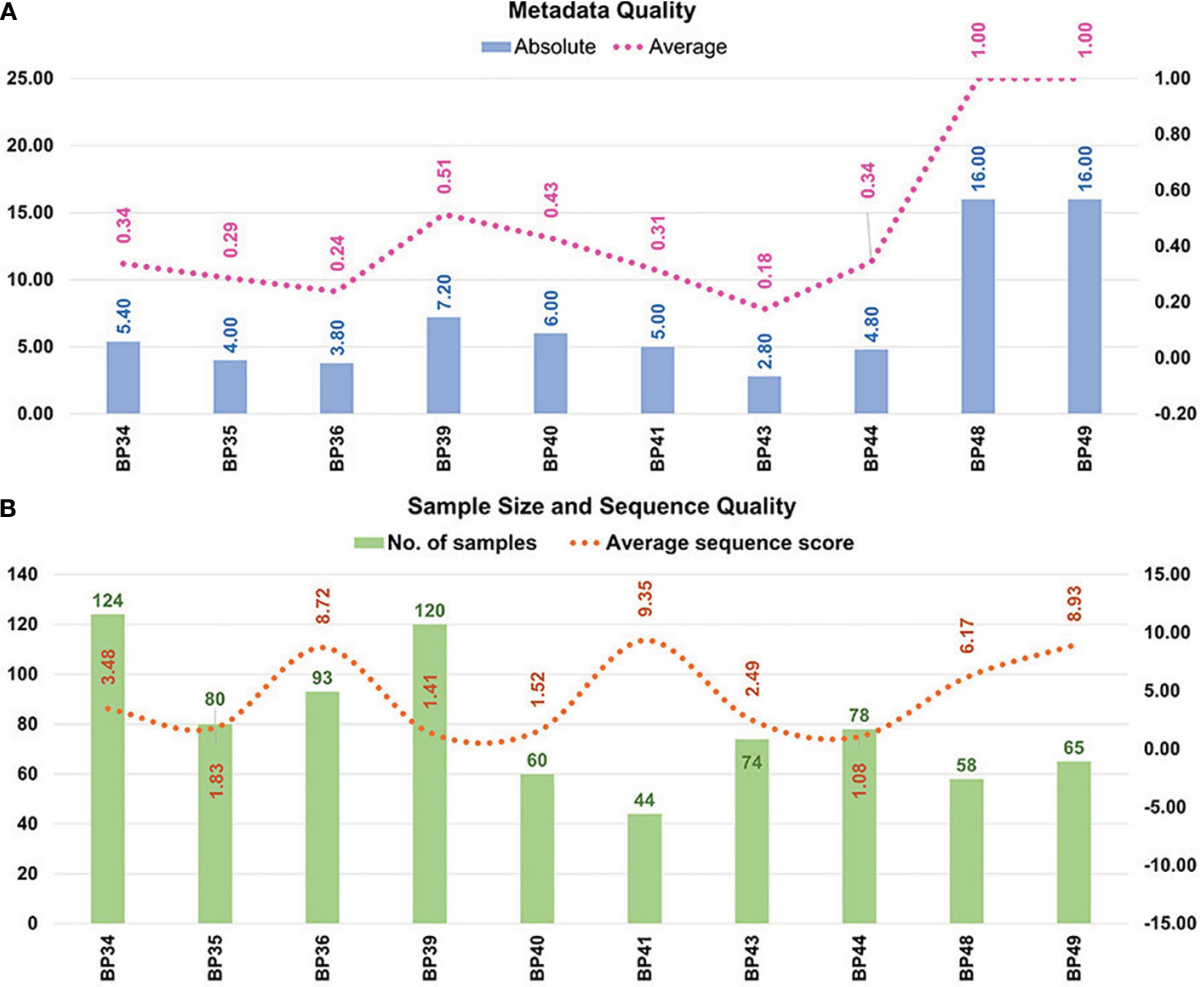
The methodological quality of the selected studies and bioprojects. Methodological quality of **(A)** metadata; and **(B)** sample size and sequence quantity. Eight articles, whose sequence data were stored in eight bioprojects, and two bioprojects associated with our samples were included in the multi-batch analysis. The 16 variables evaluated in the own-design metadata checklist were the following: 1) health condition; 2) periodontitis type and severity (if applicable); 3) sample type; 4) saliva type; 5) therapy; 6) age; 7) sex; 8) ethnicity; 9) systemic health condition; 10) smoking habit; and 11) periodontal parameters: number of teeth, bacterial plaque level, total bleeding on probing, probing pocket depth and clinical attachment level. BP34= Sun et al., 2020; BP35= Ji et al., 2020; BP36= Lundmark et al., 2019; BP39= Zhu et al., 2020b; BP40= Zhu et al., 2020a; BP41= Relvas et al., 2021; BP43= Annavajhala et al., 2020; BP44= Hall et al., 2017; BP48= own unpublished data (PRJNA774299); BP49= own unpublished data (PRJNA774981).

**Figure 4** is a graphic representation of the quality assessments of the metadata and sequences included in the 16S-MB study.

According to the metadata available on the subjects to whom the samples belonged, two of the 10 bioprojects had high-quality metadata (value of 1.00), four had medium quality (range= 0.34−0.51), and another four had low quality (range= 0.18−0.31). The bioprojects classified as medium quality lacked information about the ethnicity or clinical parameters of the study’s subjects. Those categorized as low quality also failed to include the periodontitis type and severity, as well as the participant’s age, sex, and smoking habit.

In terms of the sample size, one bioproject had <50 samples (10%), seven between 50 and 100 (70%), and two >100 (20%). Our ASS parameter analysis identified no bioprojects with values <0.25, as these had been removed in previous stages due to their very low number of sequences. Four bioprojects and 338 samples, representing 42.46% of those processed, had values from 1.00–2.00. These were regarded as high quantities, with 10,000–20,000 sequences per sample. Lastly, six bioprojects and 458 samples, representing 57.54% of those processed, had an ASS >2.00 and were thus deemed to be very high quantity, with more than 20,000 sequences per sample.

### 3.3 Differential abundance before and after the removal of BEs

**Table 1** summarizes the differential abundance results (adjusted p-value <0.01) between the periodontally healthy and periodontitis groups before and after removing the BEs. **Data Sheet 8** includes all the taxa associated with each condition and their corresponding effect size.

**Table 1:** Number of taxa with differential abundance between the healthy and periodontitis groups.

Magnitude of effect size	Before BEs removal		After BEs removal	
	No. ASVs (% detected)	No. Species (% detected)	No. ASVs (% detected)	No. Species (% detected)
**Large** **(≥0.80; ≤-0.80)**	45 (0.46%)	33 (5.76%)	19 (0.19%)	18 (3.14%)
**Medium** **(≥0.50 - <0.80; ≤-0.50 - >-0.80)**	66 (0.67%)	38 (6.63%)	41 (0.42%)	30 (5.24%)
**Small** **(≥0.20 - <0.50; ≤-0.20 - >-0.50)**	119 (1.21%)	60 (10.47%)	120 (1.22%)	67 (11.69%)
**Negligible** **(>0.00 - <0.20; <0.00 - >-0.20)**	35 (0.36%)	29 (5.06%)	10 (0.10%)	8 (1.40%)
**All**	265 (2.69%)	115 (20.07%)	190 (1.93%)	94 (16.40%)

Analyses were performed using high-abundance taxa (i.e., >0.20). The results shown are those with an adjusted p-value <0.01. The percentages of detected ASVs and species were calculated with respect to the total number of different ASVs and species detected by at least one of the groups to be compared (9,859 ASVs; 573 species). Taxa that could not be classified at the species level (“unclassified”) were counted once, meaning that the number of species detected is the minimum that could be obtained. Positive effect size magnitude thresholds correspond to disease-associated taxa and negative thresholds to health-associated taxa.

ASVs, amplicon sequence variants; BEs, batch effects; No., number.

Before the removal of the BEs, 265 ASVs belonging to 115 different species (2.69% and 20.07% of the total detected by the two groups, respectively) demonstrated statistically significant differences in their CLR abundance values between the periodontally healthy subjects and the periodontitis patients (**Table 1**). Of these, 45 ASVs from 33 species (0.46% and 5.76%, respectively) had the greatest differences between the study groups (effect size ranges: Sal_x0Hxx= -0.80− -1.33; Sal_x0Pxx= 1.70–0.80).

Conversely, after the exclusion of the BEs, a lower number of taxa showed statistically significant differences in their CLR-abundance values between the groups: 190 ASVs from 94 species (1.93% and 16.40%, respectively). Moreover, fewer taxa (19 ASVs from 18 species -0.19% and 3.14%, respectively-) had large effect sizes (ranges: Sal_x0Hxx= -0.82– -1.10; Sal_x0Pxx= 1.63–0.84) (**Table 1**).

If we compare the taxa obtained before and after removing the BEs, 148 ASVs from 75 species were common (1.50% and 13.09%, respectively), and 76 ASVs from 48 species maintained the same magnitude of effect size (0.77% and 8.38%, respectively) (**Data Sheet 8**).

#### 3.3.1 Taxa with differential abundance in health and periodontitis before and after the removal of BEs

Concerning the specific taxa that were more abundant in each periodontal condition before the removal of BEs, 39 species (i.e., all their ASVs) were associated with health and 59 with the disease. Of these, the following taxa stood out for their effect sizes (<-1.00 or >1.00): *Streptococcus oralis* subsp. *dentisani* clade 058-AV1042 and *Streptococcus sanguinis* AV228 were health-related; and *Tannerella forsythia*-AV15, *Fusobacterium nucleatum* subsp. *vincentii*-AV10, *Treponema denticola*-AV38, *Parvimonas* sp. HMT110-AV21, *Mycoplasma faucium*-AV213, *Campylobacter rectus*-AV20, *Filifactor alocis*-AV19, and *Dialister invisus*-AV68 were periodontitis-related (**Data Sheet 8**).

After removing the BEs, 39 species were associated with health and 43 with the disease, with 25 and 33 in common with those found before removal, respectively. *S. oralis* subsp. *dentisani* clade 058-AV1042 remained one of the taxa most strongly associated with health, along with *Haemophilus sputorum*-AV564 (effect sizes <-1.00). In contrast, save for *C. rectus*-AV20 and *D. invisus*-AV68, all the taxa strongly associated with periodontitis before the removal of BEs remained so, in addition to: *Fretibacterium fastidiosum*-AV97, *Peptostreptococcaceae* [XI][G-5] *saphenum*-AV129, *Peptostreptococcaceae* [XI][G-6] *nodatum*-AV189, *Peptostreptococcaceae* [XI][G-9] *brachy*-AV51, *Porphyromonas gingivalis*-AV8, *Prevotella* sp. HMT304-AV217 and *Streptococcus constellatus*-AV101 (effect sizes >1.00) (**Data Sheet 8**).

Before and after the removal of the BEs, 17 and 12 species, respectively (seven of which were common), had distinct ASVs, each associated with one of the two periodontal conditions under study. Before the exclusion of BEs, this was the case for, e.g., *Haemophilus parainfluenzae* (Sal_x0Hxx= 12 ASVs; Sal_x0Px= AV226) and *P. gingivalis* (Sal_x0Hxx= AV229; Sal_x0Pxx= 3 ASVs). However, these two species were no longer associated with both conditions after removing BEs. On the contrary, as related to the two conditions before and after the BEs removal, there were: *Actinomyces* sp. HMT172 (Sal_x0Hxx= AV577; Sal_x0Px= AV260), *Alloprevotella tannerae* (Sal_x0Hxx= AV630; Sal_x0Px= four ASVs), *Fusobacterium periodonticum* (Sal_x0Hxx= two ASVs; Sal_x0Px= AV298), and *Rothia mucilaginosa* (Sal_x0Hxx= AV48; Sal_x0Px= AV148) (**Data Sheet 8**).

Lastly, when comparing the associations between the taxa and the clinical conditions before *vs.* after the BE corrections, we found nine ASVs and 13 species related to the opposite conditions (**Data Sheet 8**).

### 3.4 Predictive models before and after the removal of BEs

#### 3.4.1 All samples

Before removing the BEs, the predictive model for distinguishing periodontal health from periodontitis that was constructed using all the study samples consisted of 16 ASVs (0.16% of the total detected by the two groups) and had an AUC of 0.944 and an ACC of 88.57% (sensitivity= 90.73%; specificity= 87.16%; PPV= 82.08%; NPV= 93.56%) (**Table 2**). These numbers worsened after the BEs were removed: the model required more ASVs (200; 2.03%), and the measures of diagnostic accuracy, save for specificity and PPV, were reduced (AUC= 0.935; ACC= 87.69%; sensitivity= 81.79%; specificity= 91.51%; PPV= 86.20%; NPV= 88.58%) (**Table 2**).

Table 2Predictive models for distinguishing health from periodontitis using all the study samples.

**Table:** 

Before BEs removal								
Model	No. ASVs (% detected)	No. Species (% detected)	AUC	SE	SP	PPV	NPV	ACC (Up-Low)
**Best^A^**	16 (0.16%)	11 (1.92%)	0.944	90.73	87.16	82.08	93.56	88.57 (90.70–86.15)
**Other^B^**	9 (0.09%)	5 (0.87%)	0.936	87.22	88.82	83.49	91.47	88.19 (90.35–85.74)
**Other^B^**	4 (0.04%)	3 (0.52%)	0.908	82.11	87.99	81.59	88.36	85.68 (88.04–83.05)

**Table:** 

After BEs removal								
Model	No. ASVs (% detected)	No. Species (% detected)	AUC	SE	SP	PPV	NPV	ACC (Up-Low)
**Best^A^**	200 (2.03%)	99 (17.28%)	0.935	81.79	91.51	86.20	88.58	87.69 (89.89–85.20)
**Other^B^**	90 (0.91%)	55 (9.60%)	0.938	83.39	89.65	83.92	89.28	87.19 (89.43–84.66)
**Other^B^**	20 (0.20%)	19 (3.32%)	0.927	80.83	88.20	81.61	87.65	85.30 (87.69–82.65)

Analyses were performed using high-abundance taxa (i.e., >0.20). The percentages of detected ASVs and species were calculated with respect to the total number of different ASVs and species detected by at least one of the groups being compared (9,859 ASVs; 573 species). Taxa that could not be classified at the species level (“unclassified”) were counted once, meaning that the number of species detected is the minimum that could be obtained.

A-”Best” was the best model obtained by the mathematical procedure implemented in the mixOmics package (Rohart et al., 2017).

B- “Other” were the models obtained from the “best” model that were created by progressively removing the predictor ASVs with the lowest contribution factor.

ACC, accuracy; ASVs, amplicon sequence variants; AUC, area under the curve; BEs, batch effects; Low, lower boundary (limits of accuracy); No., number; NPV, negative predictive value; PPV, positive predictive value; Up, upper boundary (limits of accuracy).

Both before and after the BEs removal, the reduction in the number of predictor variables of the best models by approximately half (before= 9 ASVs, 0.09%; after= 90 ASVs, 0.91%) reduced the diagnostic accuracy parameters by less than 3.55% and 2.30%, respectively (**Table 2**). Indeed, models using as few as four ASVs (0.04%) before BEs removal and 20 (0.20%) thereafter exceeded the thresholds for AUC>0.900; sensitivity and PPV >80.00%; and specificity, NPV and ACC>85.00% (**Table 2**).

#### 3.4.2 Training and test samples

Before the BEs removal, the predictive model built on the training samples (2/3 of the total= 531) to distinguish between the two periodontal conditions under study consisted of 35 ASVs (0.36%). After being validated on the test samples (1/3 = 265), the model had an AUC of 0.955 and an ACC of 88.68% (sensitivity= 86.54%; specificity= 90.06%; PPV= 84.91%; NPV= 91.19%) (**Table 3**). Again, these values worsened when the BEs were removed, with the training model requiring more ASVs (100; 1.01%) and all the diagnostic accuracy estimates becoming poorer, save for specificity, after testing (AUC= 0.947; ACC= 86.04%; sensitivity= 78.85%; specificity= 90.68%; PPV= 84.54%; NPV= 86.90%) (**Table 3**).

Table 3Predictive models for distinguishing health from periodontitis using the training and test samples.

**Table:** 

Before BEs removal									
Model	No. ASVs (% detected)	No. Species (% detected)	Samples	AUC	SE	SP	PPV	NPV	ACC (Up-Low)
**Best^A^**	35 (0.36%)	26 (4.54%)	**Train**	0.945	90.91	85.40	80.17	93.54	87.57 (90.26–84.46)
			**Test**	0.955	86.54	90.06	84.91	91.19	88.68 (92.23–84.23)
**Other^B^**	11 (0.11%)	6 (1.05%)	**Train**	0.944	89.95	86.96	81.74	93.02	88.14 (90.76–85.08)
			**Test**	0.947	88.46	91.30	86.79	92.45	90.19 (93.49–85.96)
**Other^B^**	7 (0.07%)	4 (0.70%)	**Train**	0.931	85.17	88.20	82.41	90.16	87.01 (89.75–83.84)
			**Test**	0.937	83.65	91.93	87.00	89.70	88.68 (92.23–84.23)

**Table:** 

After BEs removal									
Model	No. ASVs (% detected)	No. Species (% detected)	Samples	AUC	SE	SP	PPV	NPV	ACC (Up-Low)
**Best^A^**	100 (1.01%)	60 (10.47%)	**Train**	0.937	81.82	90.37	84.65	88.45	87.01 (89.75–83.84)
			**Test**	0.947	78.85	90.68	84.54	86.90	86.04 (89.98–81.27)
**Other^B^**	45 (0.46%)	36 (6.28%)	**Train**	0.931	81.82	89.13	83.01	88.31	86.25 (89.07–83.03)
			**Test**	0.943	78.85	89.44	82.83	86.75	85.28 (89.32–80.43)
**Other^B^**	23 (0.23%)	21 (3.66%)	**Train**	0.923	79.90	89.44	83.08	87.27	85.69 (88.55–82.42)
			**Test**	0.941	80.77	88.82	82.35	87.73	85.66 (89.65–80.85)

Analyses were performed using high-abundance taxa (i.e., >0.20). The percentages of detected ASVs and species were calculated with respect to the total number of different ASVs and species detected by at least one of the groups being compared (9,859 ASVs; 573 species). Taxa that could not be classified at the species level (“unclassified”) were counted once, meaning that the number of species detected is the minimum that could be obtained.

A-”Best” was the best model obtained by the mathematical procedure implemented in the mixOmics package (Rohart et al., 2017).

B- “Other” were the models obtained from the “best” model that were created by progressively removing the predictor ASVs with the lowest contribution factor.

ACC, accuracy; ASVs, amplicon sequence variants; AUC, area under the curve; BEs, batch effects; Low, lower boundary (limits of accuracy); No., number; NPV, negative predictive value; PPV, positive predictive value; Up, upper boundary (limits of accuracy).

The number of predictor variables in the best training models was reduced by more than a third before BEs removal (11 ASVs; 0.11%) and by more than half thereafter (45 ASVs; 0.46%). Validation of these models with the test samples revealed that the differences in diagnostic accuracy estimators concerning the best test models were less than 1.95% before deletion and 1.75% afterwards (**Table 3**). Applying the training models consisting of only seven ASVs (0.07%) before the BEs removal and 23 (0.23%) thereafter to the test samples yielded diagnostic accuracy values above the thresholds for: AUC >0.900; sensitivity and PPV >80.00%; and specificity, NPV, and ACC >85.00% (**Table 3**).

**Figure 5:**
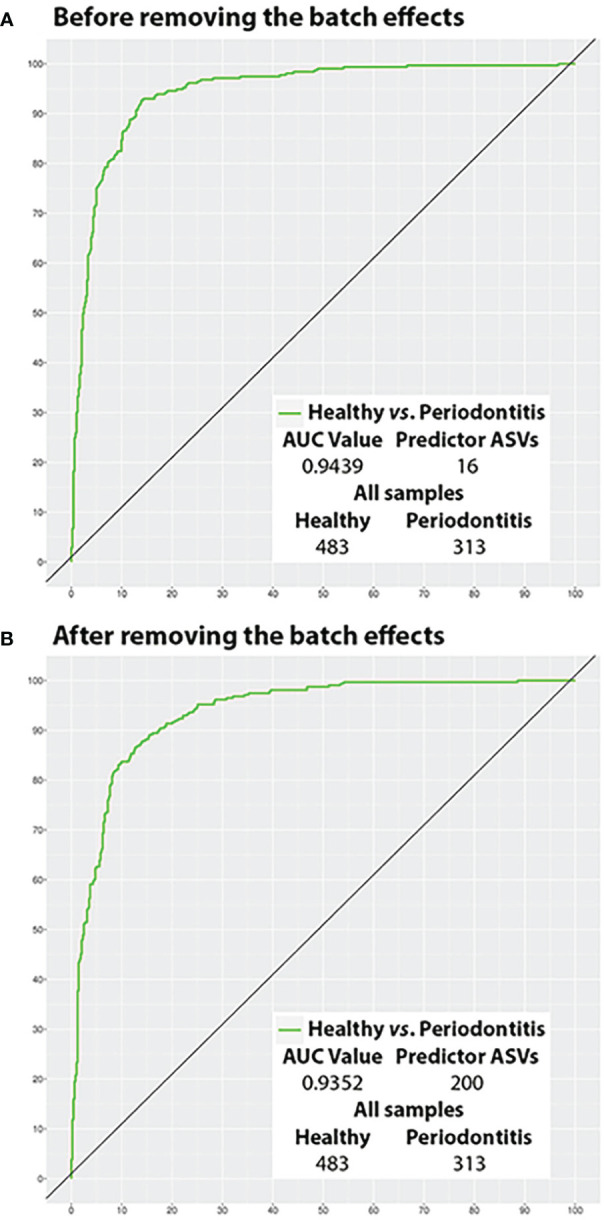
Potential of the salivary microbiota to distinguish health from periodontitis using all samples (ROC curves). **(A)** Before removing the batch effects; and **(B)** After removing the batch effects. ASVs, amplicon sequence variants; AUC, area under the curve.

**Figure 6:**
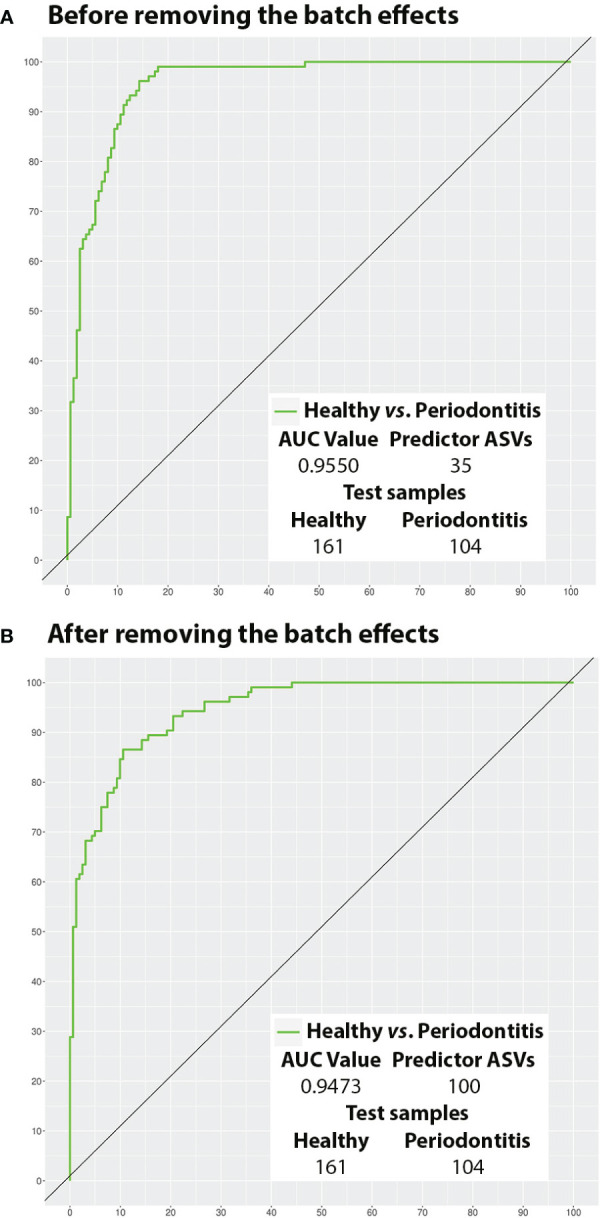
Potential of the salivary microbiota to distinguish health from periodontitis using test samples (ROC curves). **(A)** Before removing the batch effects; and **(B)** After removing the batch effects. ASVs, amplicon sequence variants; AUC, area under the curve.

The ROC curves of the best models before and after BEs removal and their AUC values are represented in **Figure 5** (All samples) and **Figure 6** (Test samples). **Data Sheet 9** contains a list of all the taxa that were part of each of the best predictive models and the periodontal condition they predicted. The diagnostic accuracy parameters obtained by all models calculated by reducing the number of predictor variables are set out in **Data Sheet 10**.

#### 3.4.3 Taxa predictive of health and periodontitis before and after the removal of BEs

**Table 4** illustrates the main taxa predictive of periodontal health and periodontitis. These were defined by focusing on taxa whose species-level taxonomy was determined and were part of the best models before removing the BEs. Moreover, taxa with a defined species-level taxonomy and a contribution value ≤-0.10 for health or ≥0.10 for disease in at least one of the best models after removing the BEs were also included.

**Table 4:** Main predictor taxa of periodontal health and periodontitis in saliva.

Taxonomy levels				Models with all samples		Models with train & test samples	
ASVid	Genus	Species	ASV	Before BEs removal	After BEs removal	Before BEs removal	After BEs removal
Predictor taxa of periodontal health in saliva							
AV00078	Haemophilus	parainfluenzae	unclassified		**-0.12**		**-0.13**
AV00382	Haemophilus	sputorum	BTASV221825		**-0.12**		**-0.12**
AV00564	Haemophilus	sputorum	unclassified		**-0.17**		**-0.18**
AV00159	Ochrobactrum	anthropi	BTASV124479			**-0.10**	
AV01019	Peptostreptococcaceae [XI][G-5]	bacterium_HMT493	BTASV114299				
AV00006	Rothia	aeria	BTASV063887		**-0.12**		**-0.12**
AV00425	Streptococcus	oralis subsp. dentisani clade 058	unclassified				
AV01042	Streptococcus	oralis subsp. dentisani clade 058	unclassified	**-0.13**	**-0.15**	**-0.20**	**-0.19**
AV00228	Streptococcus	sanguinis	unclassified			**-0.18**	
AV00571	Veillonella	rogosae	unclassified				
Predictor taxa of periodontitis in saliva							
AV00128	Alloprevotella	tannerae	unclassified		**0.13**		**0.15**
AV00061	Bacteroidales [G-2]	bacterium_HMT274	unclassified				
AV00041	Campylobacter	gracilis	BTASV155368		**0.11**		**0.14**
AV00020	Campylobacter	rectus	BTASV188427	**0.11**	**0.11**	**0.15**	
AV00068	Dialister	invisus	BTASV044355		**0.11**	**0.10**	**0.10**
AV00019	Filifactor	alocis	BTASV124203		**0.18**	**0.13**	**0.22**
AV00097	Fretibacterium	fastidiosum	unclassified		**0.18**		**0.22**
AV00044	Fusobacterium	nucleatum subsp. polymorphum	unclassified				
AV00010	Fusobacterium	nucleatum subsp. vincentii	unclassified	**0.35**	**0.17**	**0.27**	**0.18**
AV00298	Fusobacterium	periodonticum	unclassified				**0.11**
AV00213	Mycoplasma	faucium	unclassified	**0.13**	**0.17**	**0.17**	**0.21**
AV00021	Parvimonas	sp. HMT110	unclassified	**0.23**	**0.18**	**0.22**	**0.22**
AV00129	Peptostreptococcaceae [XI][G-5]	saphenum	unclassified		**0.17**		**0.22**
AV00189	Peptostreptococcaceae [XI][G-6]	nodatum	BTASV174812		**0.17**		**0.21**
AV00051	Peptostreptococcaceae [XI][G-9]	brachy	BTASV129419		**0.17**		**0.19**
AV00008	Porphyromonas	gingivalis	BTASV154369		**0.16**		**0.19**
AV00217	Prevotella	sp. HMT304	unclassified		**0.16**		**0.17**
AV00037	Sacchari	BTASV096126	unclassified		**0.10**		**0.10**
AV00237	Stomatobaculum	longum	unclassified		**0.10**		
AV00101	Streptococcus	constellatus	unclassified		**0.17**		**0.19**
AV00015	Tannerella	forsythia	BTASV153103	**0.61**	**0.23**	**0.45**	**0.30**
AV00038	Treponema	denticola	BTASV138814	**0.24**	**0.19**	**0.26**	**0.25**
AV00150	Treponema	denticola	unclassified		**0.11**		**0.12**

Cells are colored according to the periodontal condition predicted by the taxon in question: the green color is associated with periodontal health and red with periodontitis. The taxa listed in the table are those with a defined species-level taxonomy that are part of the models before removing batch effects. In addition, it also includes those with a defined species-level taxonomy and a contribution factor in at least one of the predictive models after removing batch effects: ≤-0.10 (health) or ≥0.10 (periodontitis). If no numerical value is indicated but the cell is colored, the contribution factor was >-0.10 (health) or <0.10 (periodontitis).

ASV, amplicon sequence variant; ASVid, amplicon sequence variant identifier; BEs, batch effects.

Overall, predictive taxa of periodontitis influenced the models more than those of health: 23 main disease-predictor ASVs *vs.* 10 main health-predictor ASVs. In this respect, *T. forsythia*-AV15 contributed the most in all the models. Concerning other taxa that acted as main predictors in all models, *S. oralis* subsp. *dentisani* clade 058-AV1042 is highlighted as a health predictor, and *F. nucleatum* subsp. *vincentii*-AV10, *M. faucium*-AV213, *Parvimonas* sp. HMT110-AV21, and *T. denticola*-AV38 as periodontitis predictors. Furthermore, *C. rectus*-AV20, *D. invisus*-AV68, and *F. alocis*-AV19 also predicted disease in all models, but their contribution to some of them was <0.10 (**Table 4**).

Some taxa with contribution values >-0.10 or <0.10 before maintained their low contribution or even disappeared from the models after removing BEs. Examples of this would be *S. sanguinis*-AV228 as health-predictor and *F. nucleatum* subsp. *polymorphum*-AV44 as periodontitis-predictor. Conversely, other taxa significantly increased their contribution to the models after removing BEs, such as *Rothia aeria*-AV6 for health or *P. brachy*-AV51 and *P. gingivalis*-AV8 for periodontitis (**Table 4**).

None of the main predictive taxa with contribution values ≤-0.10 or ≥0.10 appeared only in models before BEs were removed. On the contrary, others showing these values were found exclusively after correction as predictors of 1) health - *H. parainfluenzae*-AV78 and *H. sputorum*-AV382 and AV564; and 2) periodontitis - F*. fastidiosum*-AV97, *Prevotella* sp. HMT304-AV217, *Sacchari* BTASV096126-AV37, *S. constellatus*-AV101 and *T. denticola*-AV150 (**Table 4**).

In the models constructed after eliminating the BEs, there were species for which distinct ASVs predicted the two opposite clinical conditions under study, unlike the case before removal. The species affected when all samples were used were: *Actinomyces* sp. HMT172 (Sal_x0Hxx= AV577; Sal_x0Px= AV260); *A. tannerae* (Sal_x0Hxx= AV630; Sal_x0Px= four ASVs); *F. periodonticum* (Sal_x0Hxx= three ASVs; Sal_x0Px= AV298); *Neisseria perflava* (Sal_x0Hxx= three ASVs; Sal_x0Px= AV190), *Prevotella melaninogenica* (Sal_x0Hxx= AV166; Sal_x0Px= AV255) and *R. mucilaginosa* (Sal_x0Hxx= AV48; Sal_x0Px= AV148). However, this only occurred with the first two species if the train/test specimens were employed (**Data Sheet 9**).

Lastly, contrary to the differential abundance results, contrasting the condition predicted for each common ASV before *vs.* after the BEs corrections did not reveal any that predicted the opposite clinical status. Among the common species, only the *Streptococcus* unclassified to the species level predicted health before removing the BEs and both conditions thereafter (**Data Sheet 9**).

## 4 Discussion

Previous publications have evaluated the salivary microbiota of periodontally healthy and diseased patients to identify both taxa with differential abundances and those with a predictive ability to distinguish between study groups (Chen et al., 2015; Damgaard et al., 2019; Lundmark et al., 2019; Sun et al., 2020; Ma et al., 2021; Relvas et al., 2021). However, these investigations present severe methodological shortcomings related to their sample sizes, either not exceeding 60 individuals in total (Chen et al., 2015) and/or 25 in some study groups (Damgaard et al., 2019; Sun et al., 2020; Ma et al., 2021; Relvas et al., 2021), or even presenting major imbalances between groups (i.e., 25 healthy *vs.* 100 periodontitis) (Ma et al., 2021). This makes it difficult to understand and differentiate clinical conditions, as individual variations may be confounded with real biological differences (Meuric et al., 2017; Cai et al., 2021); in predictivity analyses, this leads to the obtention of overfitted and non-generalizable models (Kuhn and Johnson, 2013). Moreover, producing narrative reviews combining diverse findings to define a consensus profile for each health status is also not optimal. The methodological differences in the sequencing workflows employed in the studies can affect the diversity results (de la Cuesta-Zuluaga and Escobar, 2016; Robinson et al., 2016; Nearing et al., 2021).

Biological heterogeneity and a validation process are other methodological premises that must be fulfilled in diagnostic predictivity studies. For experts in the diagnostic accuracy field, the lack of subjects with distinct degrees of disease severity might favor overestimating a model’s accuracy parameters (Dinnes et al., 2005; Reitsma et al., 2009). A validation process, preferably external, is also needed to ensure the applicability of predictive models in other populations (Moons et al., 2015). However, some of the research published so far only included severe cases of the disease (Damgaard et al., 2019; Lundmark et al., 2019; Sun et al., 2020) and in none of them, validation of the predictive models obtained was carried out (Chen et al., 2015; Damgaard et al., 2019; Lundmark et al., 2019; Sun et al., 2020; Ma et al., 2021; Relvas et al., 2021).

These limitations can be overcome by performing 16S-MB studies in which sequences from different studies are analyzed under the same bioinformatics protocol and using the same statistical methods. This allows the analysis of enormous sample sizes, which ensures robust and reliable results (Zaura et al., 2021).

Nevertheless, before conducting a 16S-MB study, it is essential to consider the best methodological practices based on the available evidence (Regueira-Iglesias et al., 2023a). However, to our knowledge, the only 16S-MB study to date on salivary microbiota (Ruan et al., 2022) fails to meet several of these methodological practices. This study analyzed sequences from distinct gene regions and clustered them into 97% similarity OTUs to obtain taxa correlations without considering the compositionality of the data or assessing the influence of BEs.

The present is the first 16S-MB study to analyze salivary microbiota’s differential abundance and predictive capacity for identifying taxa distinguishing periodontal health from periodontitis. For the first time, these analyses on oral microbiome data were performed before and after BE removal under a CoDA analysis approach. In particular, datasets from 10 Illumina V3-V4 region bioprojects were merged, and ~800 samples were assigned to one of two clinical groups for evaluation. The patients included had different degrees of disease severity, preferable in predictive analyses since studies on only severe cases tend to overestimate diagnostic performance (Dinnes et al., 2005; Reitsma et al., 2009). A strict and unified bioinformatics protocol was applied to process the sequences. This included the employment of ASVs and an oral-specific database for taxonomic assignment (Escapa et al., 2020). In addition, external validation of the predictive models was carried out, as recommended by the TRIPOD guidelines (Moons et al., 2015).

The novel nature of our research conditioned us to compare our results with those of non-16S-MB articles that employ differential abundance and predictivity analyses (Chen et al., 2015; Damgaard et al., 2019; Lundmark et al., 2019; Sun et al., 2020; Ma et al., 2021; Relvas et al., 2021). However, based on the above arguments, these comparisons must be interpreted cautiously.

### 4.1 Quality assessment of metadata and sequences

It is critical to report metadata correctly for meaningful genomic sequence-sample environment linkage. Efforts have been made to standardize the minimum gene sequence information to be reported (Yilmaz et al., 2011). The recommended 70-variable checklist conceived for the oral environment is not yet widely used and, more importantly, has its limitations (Vangay et al., 2021). Considering this and given the limited data stored in the relevant repositories, we created a 16-variable checklist based on the minimum metadata required to perform the present 16S-MB study. This was then used to assess the quality of the metadata of the included studies.

The authors of ~38% of the articles that met the inclusion criteria had to be contacted to acquire metadata or for clarification purposes. Subsequently, as noted in a meta-analysis of the respiratory microbiome (Broderick et al., 2023), a third of these were ultimately rejected. Moreover, 80% of the included bioprojects had low- or medium-quality metadata lacking key clinical characteristics, as reported (Broderick et al., 2023). The full-text manuscripts also often had to be revised to obtain the information required.

Conversely, however, the robustness of the results of our study is guaranteed for two reasons. First, the strict quality filter was applied to the 16S rRNA gene sequences of the selected bioprojects. Second, they all had an average number of ≥10,000 sequences (range of average sequences/bioproject= 87,193-10,773).

### 4.2 Differential abundance analysis before and after the removal of BEs

Although commonly employed in non-16-MB studies for differential abundance analysis (Chen et al., 2015; Lundmark et al., 2019; Relvas et al., 2021), tools such as DESeq2 (Love et al., 2014) or LEfSe (Segata et al., 2011) should not be used. These tools do not account for compositionality and are sensitive to sparsity, leading to unacceptably high false positive rates (Gloor et al., 2017). Analyses relying on logarithmically transformed data, such as the CLR performed here, account not only for the compositionality but also the sparsity and over-dispersion that are inherent in the microbiome data (Wang and LêCao, 2020; Narayana et al., 2021). Thus, these should be performed instead (Quinn et al., 2019). On the other hand, it was also mandatory for us to perform a logarithmic transformation to apply the Wang and Lê Cao (2023) protocol for removing BEs, as some of the included approaches required it.

As anticipated, the present study’s use of analyses that remove heterogeneity from data caused by unwanted sources of variation (i.e., BEs) while also preserving the impact of real biological factors (Wang and LêCao, 2020; Narayana et al., 2021) resulted in a reduction of the total number of ASVs with differential abundance by approximately one-third. Moreover, nearly 45% of the ASVs with differential abundance between the groups before the BE removal were not retained thereafter. The taxa affected most were those with small or negligible rather than large or medium effect sizes (80 and 27 ASVs, respectively). This highlights that, as might be anticipated, most spurious associations had a low impact. These spurious associations of exposure with microbiome features are due to an imbalanced distribution between batches (Ma, 2019) and are of common appearance when pooling non-normalized samples from different studies (Gibbons et al., 2018).

#### 4.2.1 Taxa with differential abundance in health and periodontitis before and after the removal of BEs

Some of the taxa demonstrating a differential abundance about health or periodontitis, both before and after the removal of BEs, have previously been found to be more abundant in the same health conditions. Examples are the commensal species *S. oralis* subsp. *dentisani* (Sun et al., 2020) and the pathogens *F. alocis* (Lundmark et al., 2019; Diao et al., 2021; Narita and Kodama, 2022), *F. nucleatum* (Damgaard et al., 2019; Narita and Kodama, 2022), *M. faucium* (Damgaard et al., 2019; Relvas et al., 2021), *T. forsythia* (Lundmark et al., 2019; Sun et al., 2020; Diao et al., 2021; Relvas et al., 2021; Narita and Kodama, 2022) and *T. denticola* (Narita and Kodama, 2022). Similarly, others only strongly associated with periodontitis before the elimination of BEs, such as *C. rectus* (Diao et al., 2021) and *D. invisus* (Narita and Kodama, 2022), or only thereafter, such as *P. saphenum* (Damgaard et al., 2019) and *P*. HMT304 (Acharya et al., 2019), have been linked to this disease by other authors.

On the other hand, some of the contradictions we identified with the findings reported in the literature may arise because our analysis was conducted at the variant level. Consequently, we have identified how species traditionally more abundant in health, such as *R. mucilaginosa* (Narita and Kodama, 2022), have different ASVs for each condition. In line with this, removing BEs may have conditioned our discovery that species associated with a particular clinical status, such as *N. perflava* (health) (Narita and Kodama, 2022) and *P. melaninogenica* (periodontitis) (Diao et al., 2021) both have ASVs for both conditions. Conversely, after removing BEs, we confirmed the role of species widely known to be associated with health, like *C. concisus* (Lundmark et al., 2019) and *H. parainfluenzae* (Diao et al., 2021; Relvas et al., 2021) and periodontitis, such as *P. gingivalis* (Damgaard et al., 2019; Sun et al., 2020). This suggests their association with both conditions before BEs removal may have been masked by the presence of BEs.

### 4.3 Predictive models before and after the removal of BEs

In the present study, a small proportion of the salivary taxa had an outstanding ability (AUC≥0.90) (Hosmer et al., 2013) to distinguish between periodontal conditions before the removal of BEs, both in the models using all the samples (16 ASVs) and those built using training specimens and subsequently validated (35 ASVs). Moreover, these models achieved excellent sensitivity and PPV (>80%) (De Luca Canto et al., 2015) and excellent or good specificity and NPV (>85%) (De Luca Canto et al., 2015). These performance parameters are better than those provided by other predictive studies with methodological shortcomings (Chen et al., 2015; Damgaard et al., 2019; Ma et al., 2021).

After the removal of BEs, the outstanding ability of saliva in terms of AUC to distinguish health from disease was not altered. However, our all-sample and training/test-specimen models showed a decrease in all the other diagnostic classification parameters (except specificity), which did not significantly condition their interpretation (De Luca Canto et al., 2015).

Most importantly, after removing BEs, our models required around twelve (all: 200 ASVs) and three (training/test: 100 ASVs) times more predictor taxa. This latter finding contrasts the results from the differential abundance analysis, where the number of taxa fell when BEs were removed. Consistent with the contributions made by Goh et al. (2017), our predictive results showed that BEs can be confounded with hidden biological heterogeneity of the subpopulation. Consequently, when BEs are eliminated, that removal may prejudice interclass differences, creating less robust classifiers. Thus, we argue that removing BEs may not be appropriate for predictivity analysis.

On the other hand, by reducing the predictor variables in, e.g. training/test models, we observed that even using as few as four ASVs before BEs removal or 20 ASVs after, it was still possible to classify healthy and periodontitis subjects optimally. These findings corroborate the reliability, interpretability, and applicability of the models obtained (Kuhn and Johnson, 2013).

#### 4.3.1 Taxa predictive of health and periodontitis before and after the removal of BEs

*S. oralis* subsp. *dentisani* clade 058, a species commonly associated with health in the human mouth (López-López et al., 2017; Jansen et al., 2021), is described here as a health-predictor taxa in all the models. Indeed, reports have referred not only to its inhibitory activity over microorganisms traditionally considered to be oral pathogens, such as *Aggregatibacter actinomycetemcomitans*, *F. nucleatum*, *Prevotella intermedia*, *Streptococcus mutans*, and *Streptococcus sobrinus* (López-López et al., 2017; Jansen et al., 2021); but also to its ability to alkalinise the extracellular environment via the arginine deiminase system (López-López et al., 2017). Furthermore, in accordance with Diao et al. (2021), who observed that a lower abundance of *H. parainfluenzae* might be a biomarker of periodontitis, our models found that this species predicted health after removing BEs.

On the other hand, similar to that reported in non-16S-MB studies, we confirmed in all the salivary models the periodontitis-predictive role of the widely known periodontopathogens *F. alocis* (Chen et al., 2015; Lundmark et al., 2019; Ma et al., 2021), *F. nucleatum* (Lundmark et al., 2019; Ma et al., 2021), *T. forsythia* (Chen et al., 2015; Lundmark et al., 2019; Ma et al., 2021; Narita and Kodama, 2022) and *T. denticola* (Narita and Kodama, 2022). Moreover, after removing the BEs, the models found that *S. constellatus* was predictive of disease, as described in previous work (Ma et al., 2021).

As observed in the differential abundance analyses before and after BEs removal, the models after their exclusion found that different ASVs from the same species predicted the opposite clinical states. These findings indicate that the bioinformatic concept of ASV can have a real biological meaning (Callahan et al., 2017; Prodan et al., 2020; Regueira-Iglesias et al., 2023a). Consequently, associating a species-level taxon with a particular health condition might not always be appropriate.

On the other hand, unlike the differential abundance results, no ASV predicted opposite periodontal conditions before *vs.* after the removal of BEs. Consequently, in the present series, predictive modeling enabled us to better understand saliva’s health- and disease-associated taxa than the differential abundance analysis.

### 4.4 Strengths and limitations of the present study

As explained above, our 16S-MB study considered methodological best practices based on the available evidence (Regueira-Iglesias et al., 2023a).

Among the main strengths of our research were the high sample sizes of the two study groups (>450 health, >300 periodontitis) and the biological heterogeneity of their samples (i.e., different degrees of disease severity). These characteristics avoided the obtention of over-fitted and non-generalized models (Dinnes et al., 2005; Reitsma et al., 2009; Kuhn and Johnson, 2013; Papoutsoglou et al., 2023) and allowed the creation of training and test groups of sufficient size to evaluate the performance of such models (external validation). These requirements must be met in any diagnostic predictivity study (Kuhn and Johnson, 2013; Moons et al., 2015).

The employment of the same variable selection procedure and modeling technique before and after the removal of BEs allowed, for the first time in the oral microbiome literature, to evaluate the influence of such effects on the results obtained. Since the removal of BEs was assessed using five different methods (Wang and Lê Cao, 2023), the most optimal one for our data could be chosen.

Furthermore, in contrast to previous non-16S-MB articles (Chen et al., 2015; Damgaard et al., 2019; Sun et al., 2020; Diao et al., 2021; Ma et al., 2021; Relvas et al., 2021), we did not only calculate AUC values as an accuracy parameter as this is insufficient to evidence the suitability of a diagnostic biomarker (Kuhn and Johnson, 2013). We evaluated the ability to detect periodontally healthy or periodontitis patients using other classification parameters (ACC, sensitivity, specificity, PPV, and NPV).

As another advantage, we incorporated an automated procedure that, starting from the best models obtained, allowed us to identify the minimum number of predictors to obtain optimal discrimination and classification parameters.

On the other hand, one of the main limitations of our study was that, although the initial aim was to assess several periodontal conditions, we were only able to evaluate health *vs.* periodontitis. Not enough samples were detected for other conditions to perform the analyses, ensuring minimum quality standards. Finally, ~17% of the full-text excluded articles had used the Illumina technology but had not stored their sequences in public repositories. Further publications were eliminated for inadequate metadata reporting or were included but had low-quality metadata. Consequently, in line with the National Microbiome Data Collaborative Workshop report (Vangay et al., 2021), storing sequences and their corresponding metadata in public databases should be mandatory. Minimum quality standards should also be fulfilled to facilitate the reproducibility of future research and large-scale 16S-MB studies.

The findings described here contribute to advancing NGS clinical metagenomics (Chiu and Miller, 2019), offering novel insights into periodontal diagnostics. The increasing trend towards improvement in terms of cost and time of this technology could favor implementing microbiome-based diagnostic tools in daily clinical practice.

In conclusion, the removal of BEs controls false positive ASVs in the differential abundance analysis. However, their elimination implies a significantly larger number of predictor taxa to achieve optimal performance, creating less robust classifiers. As all the provided models can accurately discriminate health from periodontitis, implying good/excellent sensitivities/specificities, saliva demonstrates potential clinical applicability as a precision diagnostic tool for periodontitis.

## Data availability statement

Principal data generated or analysed during this study are included in this published article. Further supplementary information on the complete sequence and taxonomy of each detected ASVs, the count table, and metadata of all saliva samples included in the present study can be found at: https://github.com/Oral-Sciences-Research-Group/batch_effect.

## Ethics statement

The recruitment of patients for this research was conducted following the principles of the Declaration of Helsinki (revised in 2000) on human experimentation studies. Its protocol was approved by the Galician Clinical Research Ethics Committee (registration number 2018/295) and the Instituto Superior de Ciências da Saúde-Norte, CESPU (registration number 35/ CEIUCS/2019). All the participants provided their written informed consent to participate in the study.

## Author contributions

AR-I: Conceptualization, Data curation, Investigation, Methodology, Writing – original draft, Writing – review & editing. BS-R: Investigation, Methodology, Writing – original draft, Writing – review & editing. TB-P: Investigation, Methodology, Writing – original draft, Writing – review & editing. MR: Methodology, Writing – original draft, Writing – review & editing. MA-S: Methodology, Writing – original draft, Writing – review & editing. CB-C: Formal analysis, Methodology, Software, Validation, Writing – original draft, Writing – review & editing. IT: Conceptualization, Funding acquisition, Investigation, Methodology, Project administration, Resources, Supervision, Visualization, Writing – original draft, Writing – review & editing.

## Supplementary material

Supplementary Figure 1 Performance of methods for removing the batch effects in four abundance filters. PLS-DA, partial least-squares discriminant analysis; RUVIV, remove unwanted variation IV; sPLS-DA, sparse partial least-squares discriminant analysis.
