# Supplementary material for: The salivary microbiome as a diagnostic biomarker of periodontitis: a 16S multi-batch study before and after the removal of batch effects
Source: Front Cell Infect Microbiol. 2024 Jul 12;14:1405699. doi: 10.3389/fcimb.2024.1405699 (PMC11272481; doi:10.3389/fcimb.2024.1405699)
Supplement: Supplementary Figure 1 — Performance of methods for removing the batch effects in four abundance filters. PLS-DA, partial least-squares discriminant analysis; RUVIV, remove unwanted variation IV; sPLS-DA, sparse partial least-squares discriminant analysis. [file DataSheet_1.zip › 00_Data_Sheet/Data_Sheet_1.docx]

Data Sheet 1. Detailed description of the materials and methods used in this study

# Material and methods

The complete analysis protocol applied in the present study is detailed in Figure 1.

## Inclusion and exclusion criteria

The present investigation included studies on the salivary microbiota of adult patients with distinct periodontal health conditions. The V3-V4 region was targeted, and the Illumina sequencing technology was employed.

Studies were considered if, irrespective of the diagnostic criteria applied, they included a reference standard for the diagnosis of a periodontal condition based on clinical (probing pocket depth, clinical attachment level) or clinical and radiographic parameters (bone loss). Those without a reference for diagnosis and those that did not evaluate the periodontal status of a subject using at least one clinical parameter were excluded.

After applying the criteria above to the studies in the literature, we selected those where the metadata of interest per sample was properly assigned in the repository. The inclusion and exclusion standards for the stored sequences were: 1) direct and reverse sequences were acceptable, whether or not the primer pair sequence was included; 2) a minimum average length ≥350 base pairs (bps) was required for the contigs; 3) the primer sequences were aligned with the full 16S rRNA gene sequence of *Escherichia coli* J01859.1 using BLAST (Altschul *et al.*, 1990) to confirm they corresponded to the region of interest; 4) samples without primers were included if, after multiple sequences were aligned with the *E. coli* J01859.1 16S rRNA gene, they were confirmed as belonging to the region of interest; 5) bioprojects in which most of the samples had ≤7,000 sequences were rejected; and 6) bioprojects in which the samples were multiplexed or had different barcodes in each file were excluded.

## Search methods for the identification and selection of investigations

Searches were conducted in July 2021 using the electronic databases PubMed, Scopus, and Embase. The search strategy comprised two sets of terms relating to 1) periodontal health conditions, oral niches, and microbiota; and 2) the 16S rRNA gene sequencing technology (Data Sheet 2). All the searches were filtered by the publication year: 2000 to 2021 (inclusive). In Scopus and Embase, they were also filtered by the type of document (Embase: article, article in press, review); source (journal); and language (English).

Additional searches were performed within the sequence read archive (SRA) database (Leinonen *et al.*, 2011) using the terms “periodontitis”, “periodontal health”, “periodontal disease”, “peri-implantitis”, “gingivitis” and “gingival health”. This ensured we had examined all of the potential bioprojects of interest, including those published as preprints.

The manipulation of the data identified in the searches was performed using the R software (4.1.2) (R Core Team, 2022). A total of 120 searches were performed in each database and the results were stored individually. Duplicates were detected via the PubMed unique identifiers and removed.

The abstracts were analyzed computationally using seven sets of terms (Data Sheet 2). Each word in the abstract belonging to the categories “oral health”, “gene” or “microbiome” was assigned 100 points; terms from the remaining groups were given one point each. Publications with both “oral health” and “microbiome” scores ≥200 and ≥300, respectively, were selected for a subsequent manual evaluation of abstracts and full texts. The analysis of words was carried out using the packages tm (0.7-8) and natural language processing (0.2-1) (Feinerer *et al.*, 2008; Hornik, 2017).

The identifiers of the bioprojects from the selected investigations were used to access the information in the SRA database (Leinonen *et al.*, 2011) and the SRA run selector (https://www.ncbi.nlm.nih.gov/Traces/study/). At this point, two own bioprojects (PRJNA774299 and PRJNA774981) were added to the total. The information related to patients from our setting and the sequencing process of the samples obtained from them is included in Data Sheet 3.

## Classification of the metadata and sequences of the selected investigations

A new metadata table was constructed for each bioproject that included variables relating to the SRA (Leinonen *et al.*, 2011) and article identifiers, as well as the clinical and demographic characteristics of the patients contributing to each sample. To this end, we used information from the SRA metadata tables and, if necessary, from the articles or the authors.

About the sequence data, we downloaded the run accession list corresponding to the study samples of interest. The free SRA Toolkit software (SRA Toolkit Development Team) was installed in the local mode to download and store the sequences using these lists. Samples from each bioproject were stored in individual fastq files.

## Preprocessing and quality control of the sequences. Obtention of multi-batch files

The preprocessing and quality assessments of the sequences were performed with USEARCH (Edgar, 2010). Sequences were aligned and assembled, with a maximum of five mismatches and a minimum similarity of 90% deemed acceptable for the 2x250 bps; for the 2x300 bps, 10 mismatches and an 80% similarity were adequate. No more than two mismatches in the sequence of each individual primer and four in a pair were allowed. Finally, we discarded all sequences with a maximum expected error >1.0 or a minimum length <300 bps.

The multi-batch files were formed using Bash by merging all the fasta files from a given bioproject into a single file (GNU, 2020).

## Mothur pipeline

We employed the mothur pipeline (Schloss *et al.*, 2009) for ASVs, with slight modifications that included the use of the Escapa *et al*. (2020) oral-specific database for taxonomic assignment. Sequences with >400 bps were allowed, but we removed those with >8 homopolymers, which are regarded as chimeras by the VSEARCH algorithm (Rognes *et al.*, 2016), as well as those classified as unknown taxa at the highest hierarchical level. Sequences were not clustered to any level, as we aimed to identify and classify the highest number of sequences possible at the ASV level. Finally, the count table, the taxonomic hierarchy at the ASV level, the phylogenetic tree, and the metadata table were exported to R-Bioconductor (Gentleman *et al.*, 2004).

## Assessment of the methodological quality of the selected investigations

The quality of the bioprojects’ metadata was independently evaluated by two authors using a self-designed checklist, which included 16 items relating to the available data of the subjects to which the samples belonged. Each variable within a bioproject was given a value from 1.0 to 0.0: 1.0= information clearly specified in the metadata table downloaded from the repository; 0.6= data obtained from the published article; 0.3= information retrieved after contacting the authors; and 0.0= unavailable data. Then, all the values from each bioproject were added together and this number was divided by the number of applicable items. The final number represented the quality of the bioproject metadata: low= 0.00-0.33; medium= 0.34-0.66; and high= 0.67-1.00.

Moreover, also considered as quality parameters were the number of samples per bioproject and the average number of high-quality sequences per sample in each bioproject. The latter was divided by 10,000 (the number of sequences needed to obtain proportional abundance for the niche being analyzed). This parameter was assigned the name average sequence score (ASS) and its values were interpreted as: very low-quantity= <0.25; low-quantity= 0.25-0.75; acceptable-quantity= 0.75-1.00; high-quantity= 1.0-2.0; and very high-quantity sequences= >2.0.

## Statistical analysis with R-Bioconductor

The statistical analysis of the 16S rRNA gene sequencing data at the ASV level was performed using R (R Core Team, 2022) and R-Bioconductor (Gentleman *et al.*, 2004) to read the data and create a phyloseq object (phyloseq package 1.40.0) (McMurdie and Holmes, 2013). Samples with <2,500 sequences were excluded (n= 1 sample), leaving us with 814 specimens that were assigned to one of three groups according to the periodontal condition of the patients:

1) Saliva; periodontal health (Sal_x0Hxx= 483).

2) Saliva; gingivitis (Sal_x0Gxx= 18).

3) Saliva; untreated periodontitis (Sal_x0Pxx= 313).

The group Sal_x0Gxx was removed due to its low sample size for developing predictive models, leaving 796 samples for analysis. ASVs with an abundance ≤10 counts and present in ≤2 samples were also excluded (Bourgon *et al.*, 2010), meaning 9,859 ASVs remained.

We then converted the data from the phyloseq (McMurdie and Holmes, 2013) object-count matrix into percentage normalized data and applied the following abundance filters: 0.00%, 0.05%, 0.10%, and 0.20%. This meant that we obtained four different matrices in which the abundance of each taxon was above the set threshold in the total number of samples. The totals of ASVs and species for each filter were: 9,859 and 573; 1,429 and 333; 659 and 208; and 355 and 142; respectively.

In parallel, an offset of one was added to the original count matrix (all taxa, non-normalized data), i.e. a value of one was added to all the data, and a centered log-ratio (CLR) transformation was performed using the mixOmics package (6.22.0) (Rohart *et al.*, 2017). Then, analyses were performed using the CLR-transformed data matrix and, for each of them, we ran each of the abundance filters first so that all of the analyses were conducted for four abundance filters.

### Analysis for the elimination of BEs

BEs were analyzed as described by Wang and Lê Cao (2023). First, using the principal component analysis (PCA) function of the mixOmics package (Rohart *et al.*, 2017) and the Scatter_Density function of the partial least-squares discriminant analysis (PLS-DA) batch package (0.2.3) (Wang and Lê Cao, 2023), we depicted a PCA plot in combination with a density plot for each principal coordinate, with the healthy and periodontitis samples colored according to the batch (in this case, the bioproject) to which they belonged. Boxplot representations were produced to identify the variance across batches of an ASV for principal coordinates 1 and 2; density graphs were represented in each of the two PCA coordinates.

Second, using the varpart function of the vegan package (2.6-4) (Oksanen *et al.*, 2019), we evaluated the variability of the microbiome data with respect to the variability of two or more response variables before the removal of BEs. That is, we evaluated the partial variability of the true response variable (healthy and periodontitis groups) relative to the partial variability of the spurious response variables (batches). The variability assigned to each abundance filter indicated that the BEs increased with the increase in the number of taxa.

Then, we performed a redundancy ordination analysis (RDA) (van den Wollenberg, 1977) to determine how much variation in the response variables was redundant concerning the variation in the explanatory variables (ASVs). Considering the R2-adjusted value, if the variance of the residuals is much larger than that of the batches or of the intersection between the batches and the true response variable, it suggests that much of the variation in the response data can be explained by the explanatory variables.

Finally, BEs were removed using the following methods: 1) the removeBatchEffect function of the limma package (3.52.4) (Ritchie *et al.*, 2015); 2) the ComBat function of the surrogate variable analysis (sva) package (3.44.0) (Leek *et al.*, 2022); 3) a PLS-DA; 4) a sparse PLS-DA (sPLS-DA); 5) the percentile_norm functions of the PLSDAbatch package (Wang and Lê Cao, 2023); and 6) RUVIV of the remove unwanted variation (ruv) package (0.9.7.1) (Gagnon-Bartsch, 2019). The performance of each method was evaluated, with removeBatchEffect (Ritchie *et al.*, 2015) and Combat (Leek *et al.*, 2022) being the best for the different abundance filters (Image S1). The distribution of samples from subjects with periodontal health and periodontitis from the different bioprojects before and after removing the BEs were visualized using a principal component analysis (PCA) and density plot (Figure 2).

### Analysis of differential abundance

The mean difference between all the ASVs for both analysis groups was assessed using the non-parametric Mann-Whitney-Wilcoxon test. The p-value obtained was adjusted with the Benjamini-Hochberg correction using the mutoss package (0.1-13) (MuToss Coding Team *et al.*, 2023). About each ASV, we obtained its corresponding effect size, including its confidence interval and magnitude (large, medium, small, and negligible), using Cohen's d and Hedges' g statistics from the effsize package (0.8.1) (Torchiano, 2020). ASVs with an adjusted p-value <0.01 were considered to have differential abundance.

### Predictive modeling analysis

The mixOmics package (Rohart *et al.*, 2017) was used to conduct a supervised classification in the form of a sPLS-DA (Lê Cao *et al.*, 2011). This was done to facilitate the categorization of the two clinical groups and identify the ASVs that best distinguished them. Predictive models were built, initially using all the study samples, and then a subset of training specimens (2/3 of the total= 531); the latter was subsequently validated with the remaining test samples (1/3= 265). Taxa below each of the four abundance thresholds were excluded from the development of the models. The number of components in each model was determined by applying the rule of thumb K-1 (K= number of classes; here, two clinical groups). Receiver operating characteristic (ROC) curves were constructed with the true positivity rate (sensitivity) as a function of the false positivity rate (1-specificity). The following diagnostic performance parameters were calculated using the confusionMatrix function of the caret package (6.0-93) (Kuhn *et al.*, 2023): area under the curve (AUC); accuracy (ACC); sensitivity; specificity; positive predictive value (PPV); and negative predictive value (NPV).

Finally, the number of predictor variables was reduced in the models obtained using the method above (i.e., best models): five by five up to 30, and one by one below 30, until we were left with only one ASV. Every estimator of diagnostic accuracy was calculated for each number of predictors.

After evaluating the results obtained by the analyses using the four thresholds of abundance filtering, our focus is now on describing the outcomes achieved by the high-abundance taxa (>0.20 %).

# References

Altschul, S. F., Gish, W., Miller, W., Myers, E. W., Lipman, D. J. (1990). Basic local alignment search tool. *J. Mol. Biol.* 215, 403-410. doi: S0022-2836(05)80360-2

Bourgon, R., Gentleman, R., Huber, W. (2010). Independent filtering increases detection power for high-throughput experiments. *Proc. Natl. Acad. Sci. U. S. A.* 107, 9546-9551. doi: 10.1073/pnas.0914005107

Edgar, R. C. (2010). Search and clustering orders of magnitude faster than BLAST. *Bioinformatics* 26, 2460-2461. doi: 10.1093/bioinformatics/btq461

Escapa, I. F., Huang, Y., Chen, T., Lin, M., Kokaras, A., Dewhirst, F. E.*, et al.* (2020). Construction of habitat-specific training sets to achieve species-level assignment in 16S rRNA gene datasets. *Microbiome* 8, 65. doi: 10.1186/s40168-020-00841-w

Feinerer, I., Hornik, K., Meyer, D. (2008). Text mining infrastructure in R. *J. Stat. Softw.* 25, 1-54. doi: 10.18637/jss.v025.i05

Gagnon-Bartsch, J. (2019). Detect and remove unwanted variation using negative controls. R package. Version 0.9.7.1. https://CRAN.R-project.org/package=ruv

Gentleman, R. C., Carey, V. J., Bates, D. M., Bolstad, B., Dettling, M., Dudoit, S.*, et al.* (2004). Bioconductor: open software development for computational biology and bioinformatics. *Genome Biol.* 5, R80. doi: 10.1186/gb-2004-5-10-r80

GNU, P. (2020). Free Software Foundation. Bash. 5.1. http://www.gnu.org/

Hornik, K. (2017). NLP: Natural language processing infrastructure. R package. Version 0.1-11.

Kuhn, M., Wing, J., Weston, S., Williams, A, Keefer, C, Engelhardt, A., Cooper, T.*, et al.* (2023). caret: classification and regression training. R package. Version 6.0-93. https://CRAN.R-project.org/package=caret

Lê Cao, K. A., Boitard, S., Besse, P. (2011). Sparse PLS discriminant analysis: biologically relevant feature selection and graphical displays for multiclass problems. *BMC* Bioinformatics 12, 253-253. doi: 10.1186/1471-2105-12-253

Leek JT, Johnson WE, Parker HS, Fertig EJ, Jaffe AE, Zhang Y, Storey JD, Torres LC (2022). sva: surrogate variable analysis. R package. Version 3.44.0. https://bioconductor.org/packages/sva/

Leinonen, R., Sugawara, H., Shumway, M., International Nucleotide Sequence, Database Collaboration (2011). The sequence read archive. *Nucleic Acids Res.* 39, D19-D21. doi: 10.1093/nar/gkq1019

McMurdie, P. J., Holmes, S. (2013). phyloseq: an R package for reproducible interactive analysis and graphics of microbiome census data*PLoS One* 8, e61217. doi: 10.1371/journal.pone.0061217

MuToss Coding Team (Berlin, 2010), Blanchard, G., Dickhaus, T., Hack, N., Konietschke, F., Rohmeyer, K.*, et al.* (2023). Unified multiple testing procedures. R package. Version 0.1-13. https://CRAN.R-project.org/package=mutoss

Oksanen, J., Blanchet, F. G., Friendly, M., Kindt, R., Legendre, P., McGlinn, D.*, et al.* (2019). vegan: community ecology package. R package. Version 2.6-4. https://cran.r-project.org, https://github.com/vegandevs/vegan

R Core Team (2022). R: a language and environment for statistical computing. R package. Version 4.1.2. Vienna, Austria: R Foundation for Statistical Computing. https://www.R-project.org/

Ritchie, M. E., Phipson, B., Wu, D., Hu, Y., Law, C. W., Shi, W.*, et al.* (2015). limma powers differential expression analyses for RNA-sequencing and microarray studies. *Nucleic Acids Res.* 43, e47. doi: 10.1093/nar/gkv007

Rognes, T., Flouri, T., Nichols, B., Quince, C., Mahe, F. (2016). VSEARCH: a versatile open source tool for metagenomics. *PeerJ* 4, e2584. doi: 10.7717/peerj.2584

Rohart, F., Gautier, B., Singh, A., Lê Cao, K. (2017). mixOmics: an R package for ‘omics feature selection and multiple data integration. *PLoS Comput. Biol.* 13, e1005752. [doi: 10.1371/journal.pcbi.1005752](https://doi.org/10.1371/journal.pcbi.1005752)

Schloss, P. D., Westcott, S. L., Ryabin, T., Hall, J. R., Hartmann, M., Hollister, E. B.*, et al.* (2009). Introducing mothur: open-source, platform-independent, community-supported software for describing and comparing microbial communities. *Appl. Environ. Microbiol.* 75, 7537-7541. doi: 10.1128/AEM.01541-09

SRA Toolkit Development Team. Sequence Read Archive Toolkit. http://www.ncbi.nlm.nih.gov/Traces/sra/sra.cgi?cmd=show&f=software&m=software&s=software.

Torchiano, M. (2020). effsize: efficient effect size computation. R package. Version 0.8.1. https://CRAN.R-project.org/package=effsize

van den Wollenberg, A. L. (1977). Redundancy analysis an alternative for canonical correlation analysis. *Psychometrika* 42, 207-219. doi: 10.1007/BF02294050

Wang, Y., Lê Cao, K. (2023). PLSDA-batch: a multivariate framework to correct for batch effects in microbiome data. *Brief Bioinform* 24, bbac622. doi: 10.1093/bib/bbac622
