# Supplementary material for: The salivary microbiome as a diagnostic biomarker of periodontitis: a 16S multi-batch study before and after the removal of batch effects
Source: Front Cell Infect Microbiol. 2024 Jul 12;14:1405699. doi: 10.3389/fcimb.2024.1405699 (PMC11272481; doi:10.3389/fcimb.2024.1405699)
Supplement: Supplementary Figure 1 — Performance of methods for removing the batch effects in four abundance filters. PLS-DA, partial least-squares discriminant analysis; RUVIV, remove unwanted variation IV; sPLS-DA, sparse partial least-squares discriminant analysis. [file DataSheet_1.zip › 00_Data_Sheet/Data_Sheet_3.docx]

Data Sheet 3. Information on our own-recruited periodontally healthy and periodontitis patients and the sequencing process for the salivary samples obtained

# Material and methods

## Selection of study groups and collection of the saliva samples

A convenience sample of 124 eligible participants, comprising 50 periodontally healthy controls (control group) and 74 subjects affected by untreated periodontitis (periodontitis group), were recruited from 350 consecutive patients in the general population who were referred to the School of Medicine and Dentistry (Universidade de Santiago de Compostela, Spain) and the Instituto Superior de Ciências da Saúde Norte, Cooperativa de Ensino Superior, Politécnico e Universitário (CESPU, Gandra, Paredes, Portugal) between 2018 and 2019 for an assessment of their oral health status.

Patients were recruited if they fulfilled the following inclusion criteria: 1) age 26 to 79; 2) the presence of at least 10 natural teeth; 3) no previous periodontal treatment; 4) no medical history of diabetes mellitus, hepatic or renal disease, or other severe medical conditions or transmittable diseases; 5) no intake of systemic antimicrobials during the previous six months; 6) no intake of anti-inflammatory medication in the previous four months; 7) no routine use of oral antiseptics; 8) no history of alcohol or drug abuse; 9) no pregnancy or breastfeeding; 10) no presence of implants or orthodontic appliances; 11) have smoked for at least one year; and 12) have never smoked or stopped more than three years ago.

Two experienced dentists performed all the periodontal diagnoses. The bleeding on probing (BOP) and the bacterial plaque level (BPL) were recorded for the full mouth on a binary scale (presence/absence) at six sites per tooth. We also documented the probing pocket depth (PPD) and clinical attachment level (CAL) throughout the mouth, again at six sites per tooth, using a PCP-UNC 15 probe. Standardized radiographs of all the teeth were obtained to assess the alveolar bone status. The diagnosis of periodontitis was based on the clinical and radiographic information obtained. The control group included periodontally healthy patients who had: BOP ≤20%; no location with a PPD ≥4 mm; and no radiographic evidence of alveolar bone loss. The presence of periodontal health or moderate to severe generalized chronic periodontitis was established according to the clinical/radiographic information, applying previously published criteria (Tonetti *et al.*, 2018; Papapanou *et al.*, 2018).

The "smoking habit" of the participants was evaluated using a questionnaire, with information collected on its extent, i.e., non-smoker, former smoker, current smoker, time spent as a former or current smoker, and the number of cigarettes consumed per day.

The research was conducted following the principles of the Declaration of Helsinki (revised in 2000) on studies involving human experimentation (World Medical Association, 2013), and its protocol was approved by the Galician Clinical Research Ethics Committee (registration number 2018/295) and the Instituto Superior de Ciências da Saúde –Norte, CESPU (registration number 35/CE-IUCS/2019). All the participants provided their written informed consent to their involvement in the study.

Unstimulated saliva samples (2-3 milliliters -ml-) were collected from each participant using the spitting method (Navazesh and Christensen, 1982). None of the subjects had eaten or brushed their teeth for at least one hour before they provided their sample.

## 16S rRNA gene amplicon sequencing of saliva samples

Total DNA was extracted from the saliva samples using a commercial kit (MasterPure Complete DNA and RNA Purification Kit; Epicentre, Wisconsin, USA) according to the manufacturer’s instructions, albeit with minor modifications, including a mechanical disruption of bacteria (Pathogen Lysis Tube S; Qiagen, Hilden, Germany), and the addition of a lysozyme treatment (20 mg/ml at 37 ºC for 30 minutes). The isolated DNA was eluted in 50 µl of distilled and apyrogenic water, and its quality and concentration were assessed using a Nanodrop spectrophotometer (ND-2000 Spectrophotometer, Wilmington, USA). DNA samples with spectrophotometer ratios (Abs 260/280) between 1.5 and 2.0 were considered to be acceptable for inclusion in the study.

A polymerase chain reaction (PCR) amplification of the 16S rRNA gene was performed with the KAPA HiFi HotStart ReadyMixPCR Kit (Cat. No. KK2602, 7958935001; Kapa Biosystems, F. Hoffmann-La Roche Ltd, Basel, Switzerland). The V3-V4 hypervariable region was amplified as previously described (Willis *et al.*, 2018) using the following primers in a limited-cycle PCR:

V3-V4 Forward (5’-CCT ACG GGNGGC WGC AG-3’)

V3-V4 Reverse (5’-GAC TAC HVGGG TAT CTA ATC C-3’)

A set of modified primers V3-V4 F and V3-V4 R were also used. This set contained a 1-3 base pair (bp) "heterogeneity spacer" that we designed to mitigate the issues caused by low-sequence diversity amplicons.

Each PCR amplification was carried out on a total volume of 10 μl, which comprised 4 μl of DNA, 0.2-Μm from each forward and reverse primer, and a Kapa ready mix (Kapa Biosystems). The PCR conditions were modified by conducting: 1) an initial denaturation at 95°C for 3 minutes; 2) 25 three-step cycles at 95°C for 30 seconds, 55°C for 30 seconds and 72°C for 30 seconds; and 3) a final 5-minute extension at 72ºC. Water, up to a total volume of 50 μl, was added after the first PCR step. The reactions were purified using AMPure XP beads (Beckman Coulter, Brea, CA, USA) with a 0.9X (3 - 4 amplicon) ratio, according to the manufacturer’s instructions.

The PCR products were eluted from the magnetic beads with 32 μl of Buffer EB (Qiagen N.V, Hilden; Germany), with 30 μl of the eluate transferred to a fresh 96-well plate. The primers described above contain overhangs that enable the addition of full-length Nextera adapters. Barcodes are available for multiplex sequencing in a second PCR step, which produces sequencing-ready libraries. To this end, 5 μl of the first amplification was used as a template for the second PCR, with Nextera XT v2 adaptor primers added up to a final volume of 50 μl. The PCR mix and thermal profile employed for the first PCR were also used for the second, but only for eight cycles. After the second PCR, 25 μl of the final product was purified and normalized with the SequalPrep normalization kit (Invitrogen, Carlsbad, CA, USA), according to the manufacturer’s protocol. Libraries were eluted in a 20 μl volume and pooled for sequencing.

Final pools were quantified with a quantitative PCR (qPCR) using the Kapa library quantification kit for Illumina Platforms (Kapa Biosystems) on an ABI 7900HT real-time cycler (Applied Biosystems, Foster City, CA, USA). Sequencing using v3 chemistry with a loading concentration of 18 pM was performed in Illumina MiSeq (Illumina Inc., San Diego, CA, USA) with 2x300 bps reads. In all cases, 10% of the PhIX control libraries were spiked to increase the diversity of the sequenced samples.

In parallel, negative control tests of the sample-collection buffer, DNA-extraction, and PCR-amplification steps were conducted routinely under the same conditions and using reagents. One such non-template control was subjected to the library preparation and then sequenced.

The bacterial mock community as a positive control for the downstream procedures was taken from the ZymoBIOMICS Microbial Community DNA Standard (Catalog Number D6306, Zymo Research, Irvine, CA, USA), which is a mix of genomic DNA isolated from pure cultures of eight bacterial and two fungal strains. Mock DNAs were amplified and sequenced in the same way as all the other samples used in the experiment.

The sequences obtained were deposited in the sequence read archive (SRA) database (Leinonen *et al.*, 2011) under accession numbers PRJNA774299 and PRJNA774981.

# Results

## Clinical characteristics of the study groups from our setting

In our own-recruited periodontitis group, patients had a higher mean age than those in the healthy group. Also, there was a higher number of smokers in the diseased group, and they consumed more cigarettes per day and had been smoking for more months than healthy smokers. Concerning the periodontal clinical parameters, patients in the periodontitis group had significantly higher BOP, BPL, PPD, and CAL values in the full mouth records than subjects in the healthy group (Data Sheet 3.1).

Data Sheet 3.1. Age, sex, smoking habit, and clinical characteristics associated with periodontal status in our own-recruited healthy and periodontitis groups.

| **Clinical parameters** | **Study groups** | | |
| --- | --- | --- | --- |
|  | **Control**  **(n= 50)** | **Periodontitis**  **(n= 74)** | **p-value** |
| **Age (years)** | 44.14 (9.55) | 51.45 (9.98) | <0.001 |
| **Sex** |  |  |  |
| Female | 21 | 38 | NS |
| Male | 29 | 36 |  |
| **Smoking habit** |  |  |  |
| Non-smokers | 33 | 32 | 0.017 |
| Smokers | 17 | 42 |  |
| Cigarettes/day (no.) | 3.66 (6.80) | 9.34 (10.60) | 0.002 |
| Months of smoking (no.) | 60.88 (107.46) | 193.14 (194.15) | <0.001 |
| **No. of teeth** | 27.28 (2.63) | 23.68 (5.15) | <0.001 |
| **Full mouth** |  |  |  |
| BOP (%) | 10.10 (6.44) | 49.97 (21.46) | <0.001 |
| BPL (%) | 24.26 (18.62) | 52.24 (29.42) | <0.001 |
| PPD (mm) | 2.04 (0.35) | 3.65 (0.68) | <0.001 |
| CAL (mm) | 2.14 (0.44) | 4.65 (1.34) | <0.001 |

Values indicate means (standard deviations) and the number of subjects. After applying the Shapiro-Wilks test and verifying the non-normal distribution of almost all the clinical variables, the Mann-Whitney U test (two-tailed) was used to compare the quantitative clinical variables between the control and periodontitis groups. The Fisher’s exact test (two-tailed) was used to assess the association of the qualitative variables between the two study groups. A significance level of *p*<0.05 was established.

BOP= bleeding on probing; BPL= bacterial plaque level; CAL= clinical attachment level; mm= millimeters; n= sample size; No.= number; NS= No significant; PPD= probing pocket depth.

# References

Leinonen, R., Sugawara, H., Shumway, M., International Nucleotide Sequence, Database Collaboration (2011). The sequence read archive. *Nucleic Acids Res.* 39, D19-D21. doi: 10.1093/nar/gkq1019

Navazesh, M., Christensen, C. M. (1982). A comparison of whole mouth resting and stimulated salivary measurement procedures. *J. Dent. Res.* 61, 1158-1162. doi: 10.1177/00220345820610100901

Papapanou, P. N., Sanz, M., Buduneli, N., Dietrich, T., Feres, M., Fine, D. H.*, et al.* (2018). Periodontitis: consensus report of workgroup 2 of the 2017 World Workshop on the Classification of Periodontal and Peri-Implant Diseases and Conditions. *J. Periodontol.* 89 Suppl 1, S173-S182. doi: 10.1002/JPER.17-0721

Tonetti, M. S., Greenwell, H., Kornman, K. S. (2018). Staging and grading of periodontitis: framework and proposal of a new classification and case definition. *J. Periodontol.* 89, S159-S172. doi: 10.1002/JPER.18-0006

Willis, J. R., González-Torres, P., Pittis, A. A., Bejarano, L. A., Cozzuto, L., Andreu-Somavilla, N.*, et al.* (2018). Citizen science charts two major “stomatotypes” in the oral microbiome of adolescents and reveals links with habits and drinking water composition. *Microbiome* 6, 218. doi: 10.1186/s40168-018-0592-3

World Medical Association (2013). World Medical Association Declaration of Helsinki: ethical principles for medical research involving human subjects. *JAMA* 310, 2191-2194. doi: 10.1001/jama.2013.281053
