# Supplementary material for: The salivary microbiome as a diagnostic biomarker of periodontitis: a 16S multi-batch study before and after the removal of batch effects
Source: Front Cell Infect Microbiol. 2024 Jul 12;14:1405699. doi: 10.3389/fcimb.2024.1405699 (PMC11272481; doi:10.3389/fcimb.2024.1405699)
Supplement: Supplementary Figure 1 — Performance of methods for removing the batch effects in four abundance filters. PLS-DA, partial least-squares discriminant analysis; RUVIV, remove unwanted variation IV; sPLS-DA, sparse partial least-squares discriminant analysis. [file DataSheet_1.zip › 00_Data_Sheet/Data_Sheet_7.docx]

Data Sheet 7. Characteristics of the sequencing-based studies on the periodontal microbiome included in the present study

| **First author, year, bioproject** | **CPD**  **2018** | **Age** | **Sex** | **Ethnicity** | **Health condition** | **Sample** | **Periodontal therapy** | **Therapy**  **type** | **Systemic condition** | **Smoking** |
| --- | --- | --- | --- | --- | --- | --- | --- | --- | --- | --- |
|  |  | **Pt** | **Pt** | **Pt** | **Pt; Spl** | **Spl** | **Spl** | **Spl** | **Pt** | **Pt** |
| Hall *et al.*, 2017 PRJEB11529 | NO | Mean ± SD (range): 25.3 ± 3.1 (22 - 29)^A^ | F (4)^A^  M (6)^A^ | NSp (10)^A^ | H (8; 78)  G (2; 18)^B1^ | Stim sal (96)^A^ | NA | NA | Healthy (10)^A^ | NS (10)^A^ |
| Lundmark *et al.*, 2019 PRJEB21767 | NO | Mean (range): 42.7 (24 - 67) (H)^A^  Mean (range): 60.9 (22 - 88) (P)^A^ | F (68)^A^  M (46)^A^ | NSp (114)^A^ | H (47; 47)  CP (46; 46) | Stim sal (93) | NA | NA | Arthritis (1)^A^  Diabetes (3)^A^  ↑Blood press (9)^A^  Heart disease (3)^A^  NSp (98)^A^ | NSp (114)^A^ |
| Annavajhala *et al.*, 2020  PRJNA471556 | NO | Mean (IQR): 56 (52 - 61)^A^ | F (43)^A^  M (9)^A^ | Black (27)^A^  Hisp (29)^A^ | H (2; 4)  P Mild (2; 3)  P Mod (16; 26)  P Sev (22; 41)  NSp (10)^B2^ | Sal (84)^A^ | NA | NA | HIV+ (52)^A^ | CS (11)^A^  FS (5)^A^  NSp (36)^A^ |
| Ji *et al.*, 2020 PRJNA503603 | NO | NSp (58) | NSp (58) | NSp (58) | H (58; 80) | Unst sal (80) | NA | NA | Healthy (24)  H. pylori inf (34) | NS (58) |
| Sun *et al.*, 2020 PRJNA601054 | NO | Mean ± SD: 28.78 ± 7.51 (H)  Mean ± SD: 44.55 ± 14.04 (P)  Mean ± SD: 60.98 ± 9.12 (DAP)  Mean ± SD: 59.25 ± 8.61 (Met) | F (46)  M (78) | NSp (124) | H (27: 27)  P (31; 31)  DAP (46; 46)  Met (20; 20) | Unst sal (124) | NA | NA | Healthy (58)  DM2 (66) | NS (124) |
| Zhu *et al.*, 2020a  PRJNA586723 | NO | Mean ± SD: 25.40 ± 1.58 | F (5)  M (5) | NSp (5) | H (10; 60) | Stim sal (60) | NA | NA | Healthy (10) | NS (5) |
| Zhu *et al.*, 2020b  PRJNA534340 | NO | Range: 23 - 27 | F (5)  M (5) | NSp (5) | H (10; 120) | Unst sal (60)  Stim sal (60) | NA | NA | Healthy (10) | NS (5) |
| Relvas *et al.*, 2021  PRJNA623352 | NO | Range: 25 - 65^A^ | NSp (44) | NSp (44) | PG0 (17; 17)  PG1 (13; 13)  PG2 (13; 13)  PG3 (1; 1) | Unst sal (44) | NA | NA | Healthy (44) | NSp (44) |
| Own unpublished data PRJNA774299 | YES | Mean (range): 48.60 (30 - 79) | F (24) M (34) | Caucasian (58) | H (22; 22) Gen P Stage II (2; 2) Gen P Stage III-IV (34; 34) | Unst sal (58) | NA | NA | Healthy (58) | NS (28) CS (30) |
| Own unpublished data PRJNA774981 | YES | Mean (range): 48.41 (26 - 72) | F (35) M (31) | Caucasian (66) | H (28; 28) Gen P Stage II (4; 4) Gen P Stage III-IV (34; 34)^B3^ | Unst sal (66)^A^ | NA | NA | Healthy (66)^A^ | NS (37)^A^ CS (29)^A^ |

^A^Data including other clinical groups/samples not included in this analysis. ^B^Samples not included in our analysis due to ^1^low final number of samples from patients with gingivitis (n= 18), ^2^unknown periodontal health status of the study subjects and ^3^having <2500 sequences per sample.

CP: Chronic periodontitis; CPD: Classification of Periodontal Diseases; CS: Current smokers; DAP: Diabetes mellitus type 2 and periodontitis; DM2: Diabetes mellitus type 2; F: Female; FS: Former smokers; G: Gingivitis; Gen; Generalized; H: Periodontal health; Hisp: Hispanic; HIV+: positive for human immunodeficiency virus; IQR: interquartile range; M: Male; Met: Diabetes mellitus type 2, periodontitis and treated with metformin; Mod: Moderate; NA: Not applicable; NS: Non-smokers; NSp: Not specified; P: Periodontitis; PG: periodontal grade (0: none; 1: mild; 2: moderate; 3: severe); Press: pressure; Pt: Patients; Sal: Saliva; SD: Standard deviation; Sev: Severe; Spl: Samples; Stim: stimulated; Unst: unstimulated.

# Characteristics of the included investigations

Only two of the selected studies (2 own bioprojects with unpublished data/10; 20%) used the new Classification of Periodontal and Peri-implant Diseases and Conditions (Tonetti *et al.*, 2018) to establish the periodontal diagnosis. The rest of the articles (8/10; 80%) used previous classifications or authors’ criteria. More than half (6/10; 60%) compared the composition and structure of the salivary microbiota of periodontally healthy subjects with that of periodontally diseased patients, three (3/10; 30%) evaluated exclusively healthy participants, and one (1/10; 10%) only people with periodontitis. The most frequently collected type of saliva was unstimulated (5/10; 50%), followed by stimulated (3/10; 30%) or both (1/10; 10%); not having mentioned the type of saliva gathered was uncommon (1/10; 10%). Moreover, changes following therapy were not evaluated in any of the studies (10/10; 100%).

# References

Annavajhala, M. K., Khan, S. D., Sullivan, S. B., Shah, J., Pass, L., Kister, K., et al. (2020). Oral and gut microbial diversity and immune regulation in patients with HIV on antiretroviral therapy. *mSphere* 51, e00798-19. doi: 10.1128/mSphere.00798-19

Hall, M. W., Singh, N., Ng, K. F., Lam, D. K., Goldberg, M. B., Tenenbaum, H. C., et al. (2017). Inter-personal diversity and temporal dynamics of dental, tongue, and salivary microbiota in the healthy oral cavity. *NPJ Biofilms Microbiomes*. 3, 2. doi: 10.1038/s41522-016-0011-0

Ji, Y., Liang, X., Lu, H. (2020). Analysis of by high-throughput sequencing: *Helicobacter pylori* infection and salivary microbiome. *BMC Oral Health* 20, 84. doi: 10.1186/s12903-020-01070-1

Lundmark, A., Hu, Y. O. O., Huss, M., Johannsen, G., Andersson, A. F., Yucel-Lindberg, T. (2019). Identification of salivary microbiota and its association with host inflammatory mediators in periodontitis. *Front. Cell. Infect. Microbiol.* 9, 216. doi: 10.3389/fcimb.2019.00216

Relvas, M., Regueira-Iglesias, A., Balsa-Castro, C., Salazar, F., Pacheco, J. J., Cabral, C., et al. (2021). Relationship between dental and periodontal health status and the salivary microbiome: bacterial diversity, co-occurrence networks and predictive models. *Sci. Rep.* 11, 929. doi: 10.1038/s41598-020-79875-x

Sun, X., Li, M., Xia, L., Fang, Z., Yu, S., Gao, J., et al. (2020). Alteration of salivary microbiome in periodontitis with or without type-2 diabetes mellitus and metformin treatment. *Sci. Rep.* 15, 15363. doi: 10.1038/s41598-020-72035-1

Tonetti, M. S., Greenwell, H., Kornman, K. S. (2018). Staging and grading of periodontitis: framework and proposal of a new classification and case definition. *J. Periodontol.* 89 Suppl 1, S159-S172. doi: 10.1002/JPER.18-0006

Zhu, C., Yuan, C., Wei, F. Q., Sun, X. Y., Zheng, S. G. (2020a). Comparative evaluation of peptidome and microbiota in different types of saliva samples. *Ann. Transl. Med.* 8, 686. doi: 10.21037/atm-20-393

Zhu, C., Yuan, C., Wei, F. Q., Sun, X. Y., Zheng, S. G. (2020b). Intraindividual variation and personal specificity of salivary microbiota. J*. Dent. Res.* 99, 1062–1071. doi: 10.1177/0022034520917155
