# Supplementary figures and images for: The salivary microbiome as a diagnostic biomarker of periodontitis: a 16S multi-batch study before and after the removal of batch effects
Source: Front Cell Infect Microbiol. 2024 Jul 12;14:1405699. doi: 10.3389/fcimb.2024.1405699 (PMC11272481; doi:10.3389/fcimb.2024.1405699)

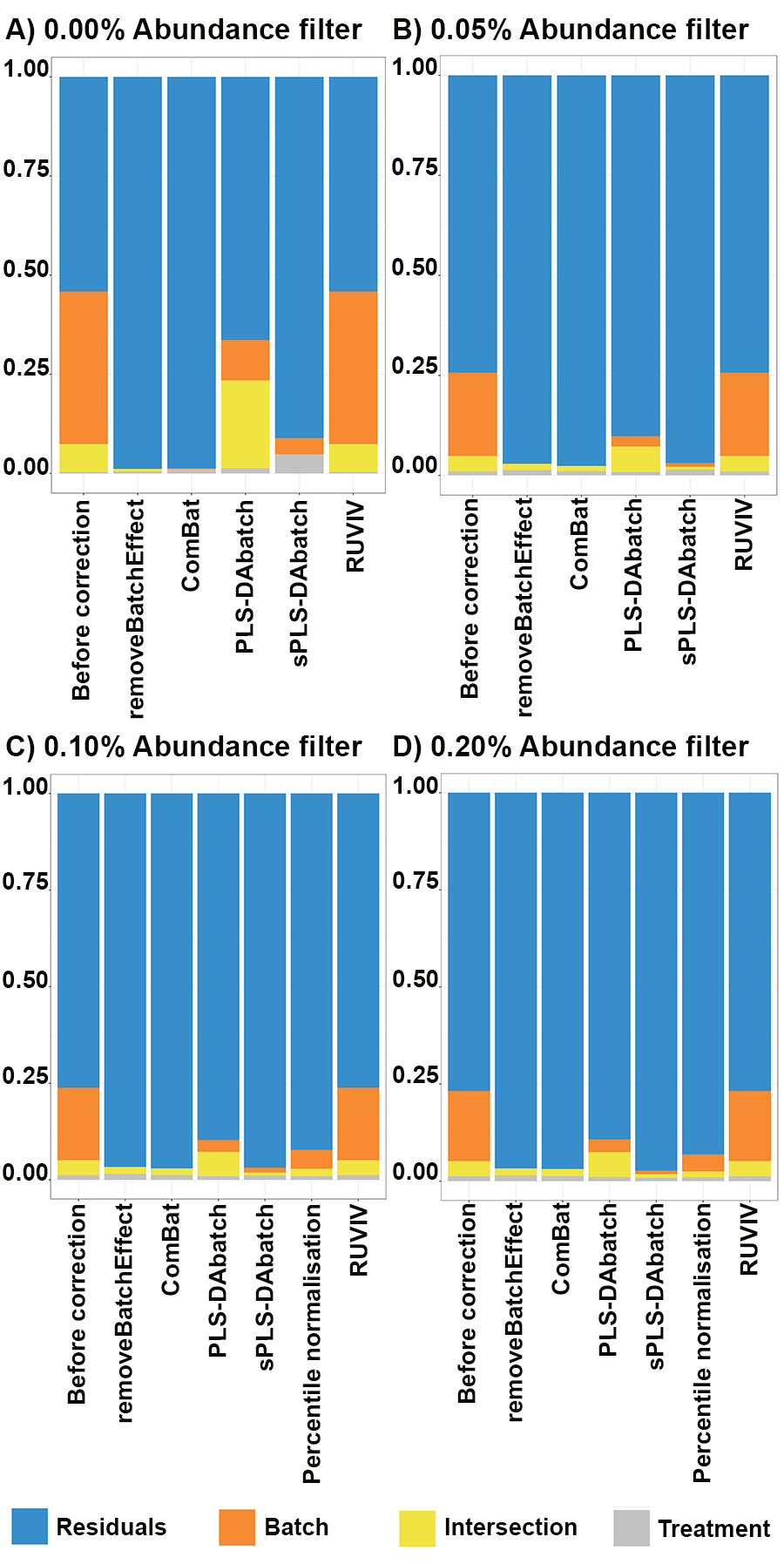

Supplement: Supplementary Figure 1 — Performance of methods for removing the batch effects in four abundance filters. PLS-DA, partial least-squares discriminant analysis; RUVIV, remove unwanted variation IV; sPLS-DA, sparse partial least-squares discriminant analysis. [file DataSheet_1.zip › 00_Data_Sheet/Image_S1.jpg]
